# Two novel diterpenoid heterodimers, Bisebracteolasins A and B, from *Euphorbia ebracteolata* Hayata, and the cancer chemotherapeutic potential of Bisebracteolasin A

**DOI:** 10.1038/s41598-017-14637-w

**Published:** 2017-11-06

**Authors:** Wen-Juan Yuan, Xiao Ding, Zhe Wang, Bi-Juan Yang, Xiao-Nian Li, Yu Zhang, Duo-Zhi Chen, Shun-Lin Li, Quan Chen, Ying-Tong Di, Haji Akber Aisa, Xiao-Jiang Hao

**Affiliations:** 1 State Key Laboratory of Phytochemistry and Plant Resources in West China, Kunming Institute of Botany, Chinese Academy of Sciences, Kunming, 650204 P.R. China; 2 Xinjiang Technical Institute of Physics and Chemistry, Chinese Academy of Sciences, Urumqi, 830011 Xinjiang P.R. China; 3 University of Chinese Academy of Sciences, Beijing, 100049 P.R. China; 4 State Key Laboratory of Biomembrane and Membrane Biotechnology, Institute of Zoology, Chinese Academy of Sciences, Beijing, 100101 P.R. China

## Abstract

Rare *ent*-abietane-rosane diterpenoid heterodimers, Bisebracteolasins A and B (**1** and **2**, respectively), were isolated from the roots of *Euphorbia ebracteolata* Hayata. Their structures and absolute configurations were elucidated from spectroscopic data and X-ray diffraction analysis. Compounds **1** and **2** exhibited moderate cytotoxic effects against five cancer cell lines. Compound **1** showed more effective antiproliferative activities against human tumour cells, HL-60 and SMMC-7721, with IC_50_ values of 2.61 and 4.08 *μ*M, respectively, than **2**. Both compounds **1** and **2** inhibit the colorectal cancer stem cell line P6C with IC_50_ values of 16.48 and 34.76 *μ*M, respectively. Moreover, preliminary biological tests showed compound **1** exhibited inhibitory activity towards tumoursphere formation and migration of the P6C cell line. Overall, we identified two novel diterpenoid heterodimers, and Bisebracteolasin A exhibits therapeutic potential in impeding tumour growth and metastatic ability of cancer stem cells.

## Introduction

Natural products (NPs) are a reliable source of and inspiration for new drugs and therapeutic agents^1–3^. Plants belonging to the Euphorbiaceae family, which consists of approximately 228 genera and 6547 species, are universally known for the chemical diversity of their isoprenoid constituents. In particular, their diterpenoids possess a variety of different core frameworks and exhibit a diverse array of beneficial activities, including anti-tumour, anti-inflammation, and immunomodulatory properties, which are industrially and scientifically attractive^4–10^.

Cancer stem cells (CSCs) refer to a small population of cancer cells that differ from the bulk tumour and play a crucial role in tumour initiation, metastasis, and drug resistance^11^. Similar to normal stem cells, all CSCs have common properties such as long lifespans, apoptosis resistance, and the capacity for self-renewal and differentiation. Notably, anti-neoplastic drugs act on more mature neoplastic cells rather than CSCs, which is explained in part by the fact that CSCs exhibit intrinsic resistance. The theory of CSCs has basic clinical implications especially because CSCs have been found in many malignant tumour tissues, and CSCs are widely considered to be more resistant to drug therapies than their differentiated progenies. The mechanism of CSCs chemoresistance involves multiple lines of defence including CSCs niches that can prevent drugs from reaching CSCs and the intrinsic quiescent or dormant property^12^. The theory of CSCs is a good explanation for tumour relapse and tumour metastasis due to the existence and sustenance of CSCs after conventional therapy, which is mediated through deregulation of multiple mechanisms and networks.

P6C, a CD44+ colorectal cancer stem cell line, was established by Prof. Chen and possesses a high tumourigenic capacity plus the characteristics of normal stem cells^13^. Traditional therapies primarily target nontumourigenic cancer cells; however, the tumour will regenerate because of the self-renewal property of cancer stem cells. Therefore, it is essential in cancer treatment to identify bioactive compounds that are capable of inhibiting both cancer and cancer stem cells^14^.

**Figure 1:**
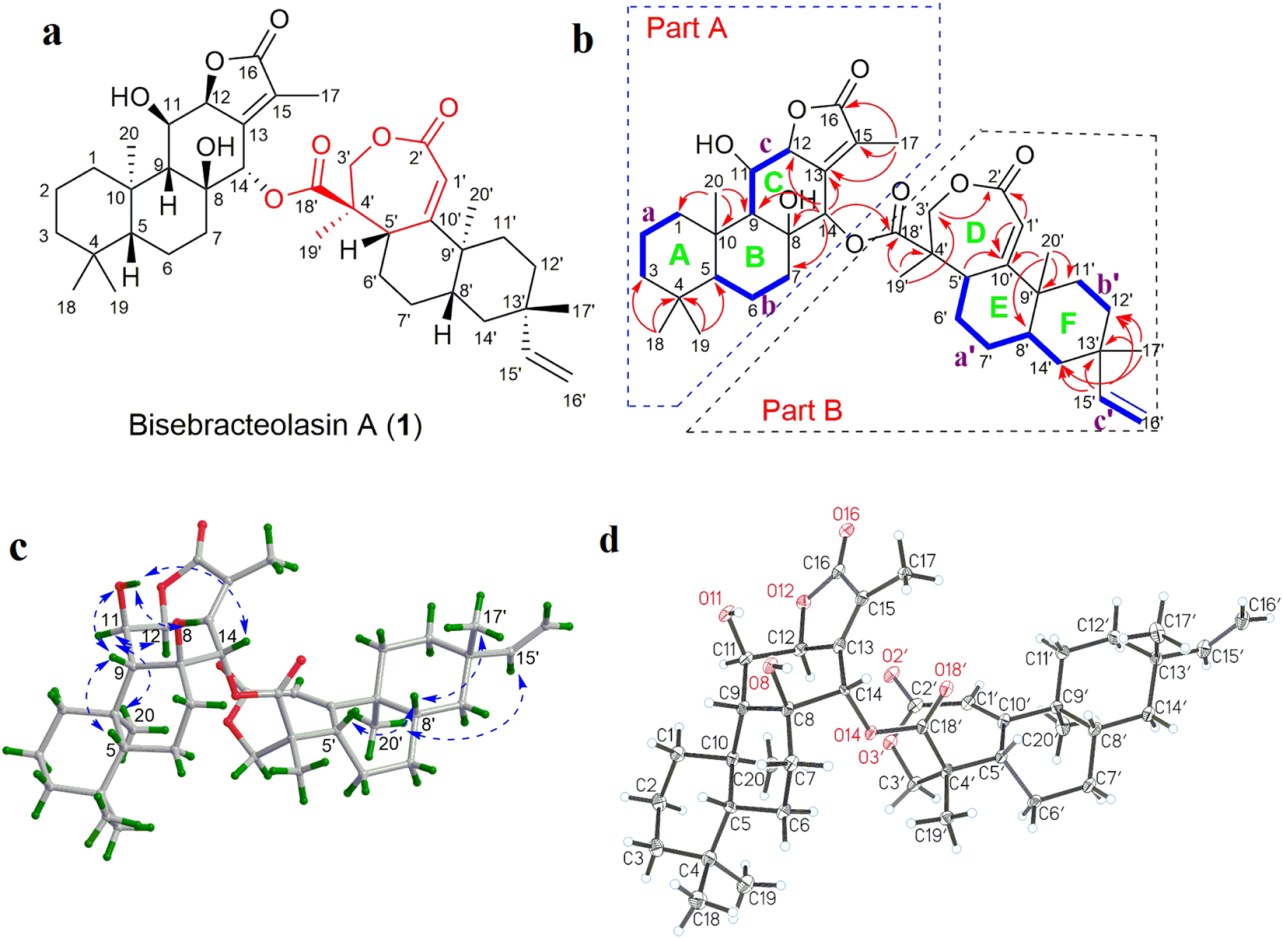
Absolute configuration of compound **1**. (**a**) Chemical structure of compound **1**. (**b**) Key ^1^H−^1^H COSY (blue) and HMBC (red arrows) correlations of compound **1**. (**c**) Key ROESY (arrow) correlations of compound **1**. (**d**) X-ray ORTEP drawing of compound **1**.

*Euphorbia ebracteolata* Hayata is a perennial herbaceous plant widely distributed in China^15^. Its root is poisonous and is used in traditional Chinese medicine for the treatment of many diseases such as cancer, swelling, and warts^16–25^. As part of our ongoing search for structurally unique and bioactive constituents from medicinal plants^26–32^, we investigated *E. ebracteolata* and discovered two new diterpenoid heterodimers featuring monomers with an unprecedented seco-2,3-rosane skeleton (Fig. 1). Herein, we describe the isolation, structural elucidation, biosynthetic pathway and bioactivity of these compounds.

## Results and Discussion

The air-dried, powdered plant material (20.0 kg) was extracted with EtOH three times, and the combined extracts were concentrated and then suspended in water. The water layer was then sequentially extracted with petroleum ether and EtOAc. The petroleum ether extract (788 g) was subjected to repeated column chromatography on silica gel, C_18_, Sephadex LH-20, and semipreparative HPLC to give two new compounds, **1** (30 mg) and **2** (15 mg).

Bisebracteolasin A (**1**) was obtained as colourless crystals with an [*α*] of −23.0 (*c* 0.2, MeOH). Its molecular formula of C_40_H_56_O_8_, which indicates 13 double-bond equivalents (DBE), was determined by HRESI(+)MS from the signal at *m/z* 687.3871 [M + Na]^+^ (calcd 687.3867). Interpretation of the ^1^H NMR spectral data (Table 1) indicated the presence of seven methyl groups (*δ*_H_ 0.85, 0.92, 1.00, 1.02, 1.18, 1.20, and 2.01), a vinylic system (*δ*_H_ 5.78, dd, *J* = 10.7, 17.5 Hz; 4.92, d, *J* = 17.5 Hz; and 4.86, d, *J* = 10.7 Hz); an olefinic proton (*δ*_H_ 6.00, d, *J* = 2.2 Hz), three oxygenated methines (*δ*_H_ 4.60, m; 5.08, s; and 5.53, s), and one oxygenated methylene (*δ*_H_ 4.22, d, *J = *12.3 Hz; and 3.96, d, *J* = 12.3 Hz). The ^13^C NMR data (Table 1) and HSQC spectrum revealed 40 carbon resonances, which were attributed to seven methyl carbons (*δ*_C_ 9.0, 13.6, 18.3, 19.9, 22.0, 22.5 and 34.0), a vinyl group (*δ*_C_ 109.5 and 150.1), three ester carbonyl groups (*δ*_C_ 168.4, 173.6, and 174.1), two olefinic groups (*δ*_C_ 167.3, 150.5, 131.0, and 115.8), five oxygenated carbon atoms (including one quaternary, three methines and one methylene), and 19 aliphatic carbon atoms (including five quaternary, four methines and ten methylenes). Analysis of the aforementioned spectroscopic data and comparison to the spectroscopic data of simultaneously isolated Yuexiandajisu D (**3**)^33^, suggested that **1** was probably a heterodimeric C-20 type diterpenoid with **3** as one of its monomers. In addition to the NMR observation, two daughter ions at *m/z* 331 and 287 in the MS/MS analysis provided further evidence for the dimeric structure.

**Table 1:** NMR Data for compounds **1** and **2** in CDCl_3_ (*δ* in ppm, *J* in Hz).

No.	1	2		
	*δ*_H_	*δ*_C_	*δ*_H_	*δ*_C_
1	a 2.03 (m)	39.7 (t)	a 2.03 (m)	39.5 (t)
	b 1.77^*a*^		b 1.70^*a*^	
2	a 1.76^*a*^	20.3 (t)	a 1.71^*a*^	20.1 (t)
	b 1.65 (m)		b 1.58^*a*^	
3	a 1.46 (m)	41.4 (t)	a 1.45 (m)	41.4 (t)
	b 1.20 (m)		b 1.20 (m)	
4	—	33.3 (s)	—	33.2 (s)
5	1.07 (m)	54.7 (d)	1.05 (m)	54.7 (d)
6	1.53^*a*^	18.7 (t)	1.51 (m)	18.7 (t)
7	a 1.94^*a*^	41.4 (t)	a 1.94^*a*^	41.4 (t)
	b 1.56^*a*^		b 1.58^*a*^	
8	—	74.0 (s)	—	74.3 (s)
9	2.12 (s)	62.0 (d)	2.09 (s)	62.0 (d)
10	—	37.7 (s)	—	37.7 (s)
11	4.60 (m)	68.3 (d)	4.57 (d, 3.5)	68.4 (d)
12	5.08 (s)	78.7 (d)	5.13 (s)	78.9 (d)
13	—	150.5 (s)	—	151.5 (s)
14	5.53 (s)	75.4 (d)	5.65 (s)	74.0 (d)
15	—	131.0 (s)	—	130.2 (s)
16	—	174.1 (s)		174.3 (s)
17	2.01 (d, 1.7)	9.0 (q)	2.03 (d, 1.8)	9.1 (q)
18	0.92 (s)	34.0 (q)	0.92 (s)	34.0 (q)
19	0.85 (s)	22.0 (q)	0.85 (s)	22.0 (q)
20	1.18 (s)	18.3 (q)	1.20 (s)	18.2 (q)
8-OH	3.61 (s)			
11-OH	4.35 (s)			
1′	6.00 (d, 2.2)	115.8 (d)	5.95 (s)	116.9 (d)
2′	—	168.4 (s)	—	171.4 (s)
3′	a 4.22 (d, 12.3)	70.8 (t)	—	177.2 (s)
	b 3.96 (d, 12.3)		—	
4′	—	50.2 (s)	—	45.9 (s)
5′	3.40 (m)	44.6 (d)	1.94^*a*^	41.5 (d)
6′	1.44 (m)	21.1 (t)	1.43 (m)	26.2 (t)
	1.07 (m)		1.02 (m)	
7′	a 1.50 (m)	24.4 (t)	a 1.59 (m)	27.5 (t)
	b 1.28^*a*^		b 1.35^*a*^	
8′	1.83^*a*^	32.5 (d)	1.72^*a*^	40.4 (d)
9′	—	40.8 (s)	—	40.9 (s)
10′	—	167.3 (s)	—	172.2 (s)
11′	a 1.71^*a*^	34.7 (t)	a 1.62^*a*^	33.7 (t)
	b 1.56^*a*^		b 1.56^*a*^	
12′	a 1.45^*a*^	32.5 (t)	a 1.61^*a*^	32.5 (t)
	b 1.35^*a*^		b 1.36 (m)	
13′	—	36.1 (s)	—	36.0 (s)
14′	a 1.28^*a*^	39.2 (t)	a 1.32 (m)	39.2 (t)
	b 1.16 (m)		b 1.02 (m)	
15′	5.78 (dd, 10.7, 17.5)	150.1 (d)	5.78 (dd, 10.7, 17.5)	150.3 (d)
16′	a 4.92 (d, 17.5)	109.5 (t)	a 4.92 (d, 17.5)	109.2 (t)
	b 4.86 (d, 10.7)		b 4.86 (d, 10.7)	
17′	1.00 (s)	22.5 (q)	1.00 (s)	22.7 (q)
18′	—	173.6 (s)	1.30 (s)	24.5 (q)
19′	1.20 (s)	13.6 (q)	1.31 (s)	27.3 (q)
20′	1.02 (s)	19.9 (q)	0.96 (s)	15.8 (q)

^*a*^Overlapped, multiplicity could not be determined.

The gross structure of **1** was constructed by 2D NMR analysis, in which the proton-bearing structural units a-c in part A and a′-c′ in part B (drawn with bold bonds) were readily established by analysis of the ^1^H- ^1^H COSY spectrum (Fig. 1b). The HMBC correlation networks of H_3_-18(19)/C-3, C-4, and C-5; H_3_-20/C-1, C-9, and C-10; H_3_-17/C-13, C-15, and C-16; H-14/C-7, C-8, C-9, C-12, and C-13, as depicted with arrows from H to C, supported the connection of A-C rings in part A, and thus confirmed Yuexiandajisu D (**3**), an *ent*-abietane-type diterpenoid, as one monomer. In view of the remaining chemical shifts, the HMBC correlations of H_3_-17′ and H-15′/C-12′, C-13′, and C-14′; H_3_-20′/C-8′, C-9′, C-10′, and C-11′; and H-5′/C-10′ (as shown) established rings E and F in part B and placed the vinyl group at C-13′. Moreover, the cross peaks of H_3_-19′/C-3′, C-4′, and C-18′; H-1′/C-2′ and C-10′; and H_2_-3′/C-2′ in the HMBC spectrum indicated that ring D was a seven-membered lactone with a double bond between C-1′ and C-10′ and a methyl and a carbonyl at C-4′. This confirmed that fragment B is a novel 2,3-seco-rosane-type diterpenoid with a 7/6/6 ring system. The two monomeric parts, A and B, were finally linked with an ester bond between C-14 and C-18′ by the key HMBC cross peak of H-14 to C-18′, which satisfied the DBE requirement of **1**. Thus, the planar structure of **1** was established to be an unprecedented 6/6/6/5/7/6/6 heptacyclic heterodimeric diterpenoid (Fig. 1b).

The relative configuration of **1** was deduced by the analysis of ROESY data and 3D computer modelling. In Part A, the ROE correlations of H-5/H-9; H-9/OH-11; and OH-11/H-14 and OH-8 indicated that the groups are cofacial, and they were arbitrarily assigned as *β*-oriented. In turn, the ROE correlations of H_3_-20/H-11*α* and H-11*α*/H-12 suggested the *α*-orientation of these groups. In Part B, the ROE correlations of H-5′/H-8′ and H-8′/H_3_-17′ indicated that the groups are cofacial, and they were arbitrarily assigned as *β*-oriented. In turn, the ROE correlation of H_3_-20′/H-15′ suggested the *α*-orientation of these groups (Fig. 1c).

The absolute configuration of **1** was finally determined via single-crystal X-ray diffraction by using the anomalous dispersion of CuK*α* radiation Fig. 1d; Flack parameter = 0.1(3); the Hooft parameter is 0.11(10) for 2372 Bijvoet pairs^34,35^. As shown in Fig. 1d, the absolute configuration of **1** was unambiguously assigned as 5 R,8 S,9 R,10 R,11 R,12 S,14 S,4′R,5′S,8′S,9′R,13′S.

**Figure 2:**
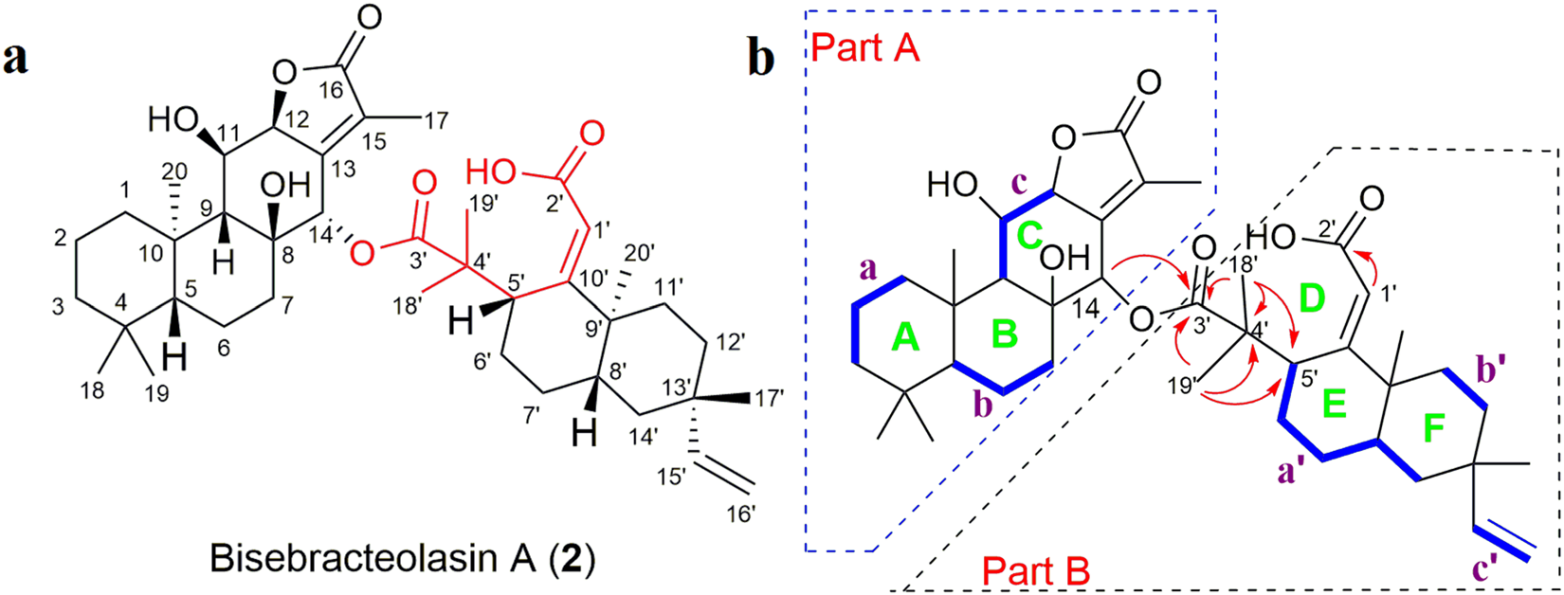
Structure of compound **2**. (**a**) Chemical structure of compound **2**. (**b**) Key ^1^H−^1^H COSY (blue) and HMBC (red arrows) correlations of compound **2**.

Compound **2** was isolated as an amorphous white powder. According to the HRESIMS data, its molecular formula is C_40_H_58_O_8_, which indicates 12 double-bond equivalents (DBE); one less than that of **1**. The ^1^H and ^13^C NMR spectra of **2** (Table 1) were very similar to those of **1** except for the absence of the resonances for the 2′,3′-lactone moiety. Instead, the spectra exhibited signals for an extra carboxyl group (*δ*_C_ 171.4) and an extra methyl (*δ*_H_ 1.30, s; *δ*_C_ 24.5). Those signals were assigned to 2′ and 18′, respectively, based on the HMBC correlations of H_3_-18′ with C-3′, C-4′ and C-5′; and of H-1′ with C-2′ (Fig. 2b). Moreover, based on the HMBC correlations of H-14 with the ester carbonyl C-3′, it was apparent that the C-2′−C-3′ bond that was present in **1** had been cleaved and replaced with a C-3′−C-14 ester bond. The ROE correlation of H-1′ (*δ*_H_ 5.95, s)/H-11′α (*δ*_H_ 1.61, m) suggested the double bond of C-1′−C-10′ is in the *E*−configuration. In addition, based on ^13^C NMR shifts and NOE data, the relative configuration of the remaining chiral centres of **2** are analogous to those of **1**. Thus, the structure of **2** (Bisebracteolasin B) was assigned as shown.

**Figure 3:**
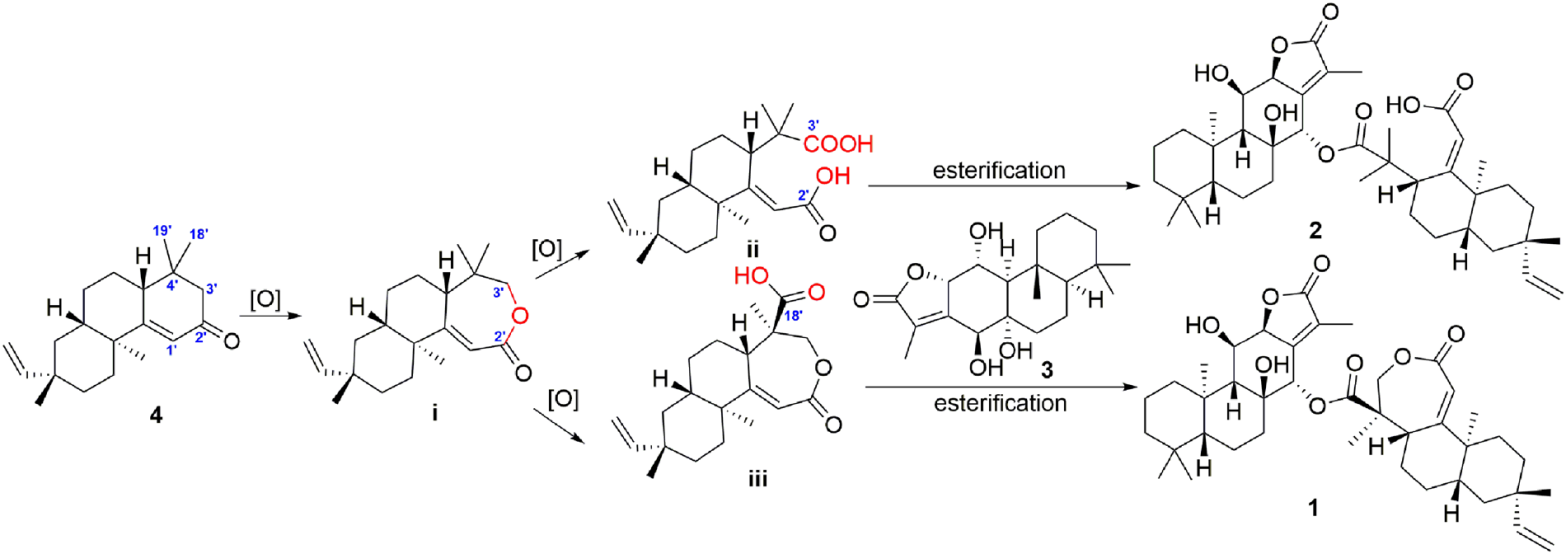
Proposed biogenetic pathway of compounds **1** and **2**.

To the best of our knowledge, compounds **1** and **2** are rare examples of diterpenoid heterodimers from the family Euphorbiaceae^36^. Plausible biosynthetic pathways to **1** and **2** are postulated (Fig. 3). Compound **4**, a proposed biogenetic precursor, could be oxidized to yield the key intermediate lactone **i**. Then, intermediate **i** could be further transformed to **ii** via oxidative cleavage. Meanwhile, subsequent oxidation of C-18′ of intermediate **i** would yield intermediate **iii**. Finally, both intermediates **ii** and **iii** could react with the co-isolated diterpenoid Yuexiandajisu D (**3**) to give **1** and **2**, respectively.

**Figure 4:**
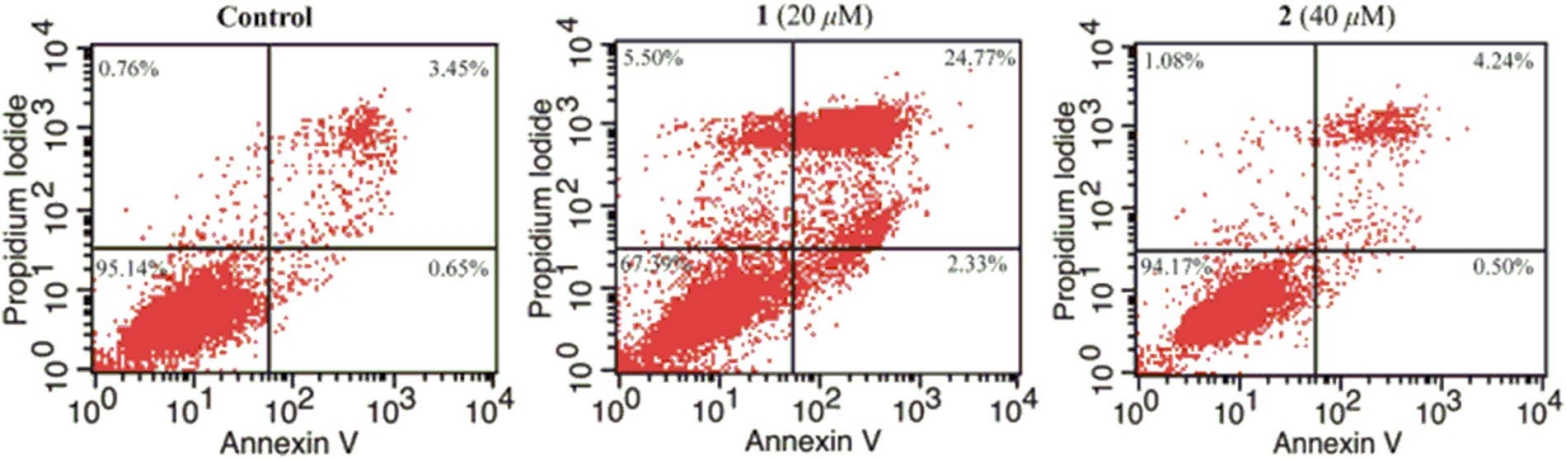
The apoptosis effects of compounds **1** and **2** on SMMC-7721.

Compounds **1** and **2** were tested for their *in vitro* cytotoxic activities against five human cancer cell lines, namely, lung cancer cell line A549, promyelocytic leukaemia cell line HL-60, breast cancer cell line MCF-7, liver cancer cell line SMMC-7721, and colon cancer cell line SW-480 using the MTS method^37^. Cisplatin and Taxol were used as the positive controls. Compound **1** showed more potent antiproliferative activities against all five cell lines than the conventional chemotherapeutic reagent cisplatin, especially in HL-60, SMMC-7721 and MCF-7 human tumour cells with IC_50_ values of 2.61* μ*M, 4.08 *μ*M and 8.17* μ*M (Table 2), respectively, compared to cisplatin which showed IC_50_ values of 19.11* μ*M, 17.72 *μ*M and 19.65* μ*M, respectively. Compound **2** showed similar antiproliferative activities as those of cisplatin. To further test the effects of compound **1**, we used Annexin V to check for apoptosis induced by compounds **1** and **2**. We found that compound **1** could induce significant apoptosis at 20 *μ*M in the SMMC-7721 cell line using flow cytometry, but compound **2** did not induce significant apoptosis (Fig. 4).

**Table 2:** Cytotoxicity of compounds **1** and **2** (IC_50_, *μ*M).

Compound	HL-60	A549	SMMC-7721	MCF-7	SW-480
**1**	2.61	10.97	4.08	8.17	10.20
**2**	11.87	11.49	10.64	16.05	14.09
DDP	19.11	12.46	17.72	19.65	11.87
Taxol	<0.008	<0.008	<0.008	<0.008	<0.008

DDP(cisplatin) and Taxol were used as positive controls.

**Figure 5:**
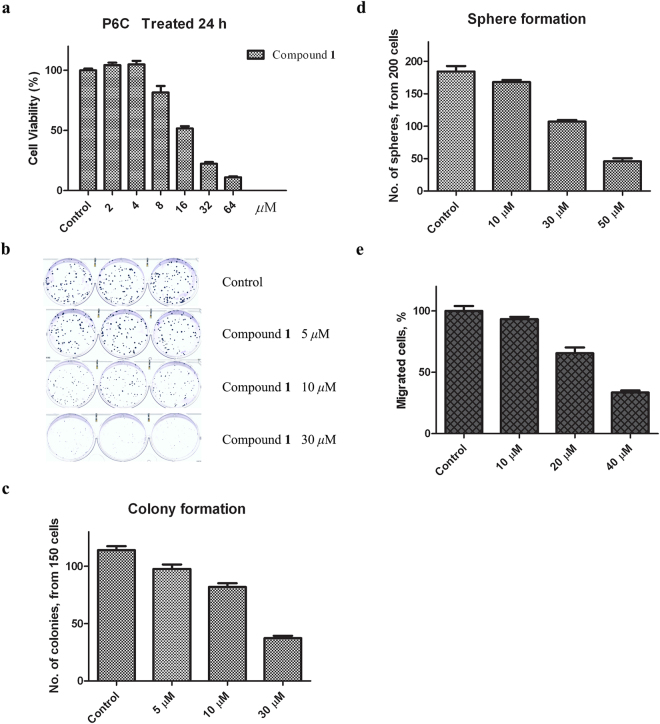
Inhibition of cell growth and migration of P6C cells by compound 1. (**a**) Compound **1** inhibited the cell growth of P6C cells. P6C cells were treated with compound **1** for 24 hours with increasing doses as indicated. The MTT viability assay was used to measure the cytotoxic effects of compound **1**. (**b**,**c**) Compound **1** inhibited the colony forming ability of P6C cells. Approximately 150 P6C cells were seeded in the wells of a 6-well plate, and compound **1** was added at different concentrations. After two weeks, the cells were fixed and stained with Coomassie Brilliant Blue R250 and counted. Representative images of the plates are shown (**b**), and the number of colonies is shown in the bar graph (**c**). (**d**) Compound **1** inhibited the sphere-forming ability of P6C cells. Approximately 200 P6C cells were seeded in 0.3% soft agar for the anchorage-independent sphere-forming assay. After three weeks, the spheres were fixed, stained and counted. The number of spheres is shown in the bar graph. (**e**) Compound **1** inhibited the migration ability of P6C cells. P6C cells were seeded in the upper layer of the well. Migrated cells on the bottom side of transwell cultures were fixed and stained. Images were taken in 5 random microscopic fields using a light microscope. In addition, the migrated cells were quantified with ImageJ. All the experiments were performed in triplicate and repeated three times with similar results. Bars represent the mean ± SD.

To evaluate the cytotoxic effects of compounds **1** and **2** toward cancer stem cells, we tested compounds **1** and **2** against the established colon cancer stem cell line P6C. Compounds **1** and **2** shown in inhibitory activity against the CD44^+^ colorectal cancer stem cell line P6C with IC_50_ values of 16.48 and 34.76 *μ*M, respectively, using MTT assays. Compound **1** caused growth inhibition of P6C in a dose-dependent manner, as shown in Fig. 5a. To check the inhibitory effect of compound **1** in P6C cells relative to its tumourigenic and metastatic potential, both anchorage-dependent and anchorage-independent assays (the colony formation assay and the soft agar assay) were employed. The inhibitory effect of compound **1** on anchorage-dependent colony formation and anchorage-independent sphere formation of P6C cells were determined two weeks after cell seeding. With the tested compounds, fewer and smaller colonies were formed on the plate than in soft agar. Consistent with the MTT assay, compound **1** caused growth inhibition in P6C cells in a dose-dependent manner (Fig. 5b,c and d). Metastasis is another hallmark of malignant tumours. We checked the effect of compound **1** on cell migration ability by *in vitro* Boyden chamber motility assays (Fig. 5e). Compound **1** greatly inhibited the migration ability of P6C cells. Moreover, compound **1** could decrease the migration ability of P6C cells in a dose-dependent manner.

In summary, Bisebracteolasins A and B (**1** and **2**) are rare oxygen-bridged heterodimeric diterpenoids, and they possess a monomer with a unique seco-2,3-rosane-type skeleton. Compound **1** exhibited significant inhibitory activity against five different cancer cells lines from different tissues and against colorectal cancer stem cells. Our results indicate that compound **1** has great potential to be developed as a therapeutic agent towards cancer cells, especially cancer stem cells. The unprecedented structures of these two compounds will trigger more in-depth pharmaceutical investigations.

## Methods

### General experimental procedures

Crystal data were acquired on a Bruker APEX DUO diffractometer with graphite monochromator CuK*α* radiation. Melting points were obtained using a Mettler Toledo MP50 micro melting point apparatus. Optical rotations were measured with a Horiba SEPA-300 polarimeter. IR spectra were obtained on a Tensor 27 spectrophotometer with KBr pellets. UV spectra were obtained using a Shimadzu UV-2401A spectrophotometer. 1D (^1^H, ^13^C, and DEPT) and 2D (^1^H-^1^H COSY, HSQC, HMBC, and ROESY) NMR spectra were collected on a Bruker Avance III 500 spectrometer. ESIMS and HRESIMS spectra were acquired on an Agilent G6230 spectrometer. Semi-preparative HPLC separations were performed on an Agilent 1100 liquid chromatograph with a Waters X-Bridge Prep Shield RP_18_ (10 × 150 mm) column. Column chromatography (CC) was performed using silica gel (100–200 and 200–300 mesh, Qingdao Marine Chemical Co. Ltd., Qingdao, China) and Sephadex LH-20 (40–70 *μ*m, Amersham Pharmacia Biotech AB, Uppsala, Sweden). Fractions were monitored by TLC (GF_254_, Qingdao Marine Chemical Co. Ltd., Qingdao, China), and spots were visualized by heating silica gel plates sprayed with 5% H_2_SO_4_ in EtOH. All solvents were distilled prior to use.

### Plant Material

The roots of *Euphorbia ebracteolata* Hayata. were collected from Anhui Province, People’s Republic of China, in November 2014. The plant samples were identified by Ji-Ming Xv of the National Institutes for Food and Drug Control. A voucher specimen (HXJ20141108) was deposited at the State Key Laboratory of Phytochemistry and Plant Resource in West China, Kunming Institute of Botany, Chinese Academy of Science (CAS).

### Extraction and Isolation

The air-dried, powdered plant material (20 kg) was extracted with 95% EtOH (3 × 50 L) under reflux three times (4, 3, and then 3 h). The combined EtOH extracts were concentrated under vacuum to give a crude residue (2.1 kg), which was suspended in water and then partitioned with petroleum ether. The petroleum ether portion (788 g) was purified on a silica gel column eluted with a gradient of petroleum ether-acetone (from 1:0 to 0:1) to yield five major fractions (1–5). Fr.4 (42 g) was then separated using a C_18_ silica gel column (MeOH-H_2_O from 4:6 to 10:0) to obtain five further fractions (4A-4E). Fr.4 C (11 g) was chromatographed on a silica gel column eluted with petroleum ether-acetone (50:1 to 5:1) to afford five subfractions (Fr4C1-Fr4C5). Fr4C2 (3 g) was purified by Sephadex LH-20 chromatography with methanol and then separated by semi-preparative HPLC (CH_3_CN-H_2_O, 5:5) to obtain **1** (30 mg). Fr4C3 (3.2 g) was purified by Sephadex LH-20 chromatography with acetone and then chromatographed on a silica gel column (CHCl_3_-Me_2_CO, 20:1) to obtain **2** (15 mg).

### MTS Assay

The following human tumour cell lines were used: HL-60, SMMC-7721, A-549, MCF-7, and SW-480. All cells were cultured in RPMI-1640 or DMEM (HyClone, Logan, UT, USA) supplemented with 10% foetal bovine serum (FBS) (HyClone) at 37 °C in a humidified atmosphere with 5% CO_2_. Cell viability was assessed by conducting colourimetric measurements of the amount of insoluble formazan formed in living cells based on the reduction of 3-(4,5-dimethylthiazol-2-yl)-5(3-carboxymethoxyphenyl)-2-(4-sulfopheny)-2H-tetrazolium (MTS) (Sigma, St. Louis, MO, USA). Briefly, 100 *μ*L of adherent cells was seeded into each well of a 96-well cell culture plate and allowed to adhere for 12 h before the addition of test compounds; however, suspended cells were seeded just before the addition of test compounds. All cells were seeded at an initial density of 1 × 10^5^ cells/mL in 100 *μ*L of medium. Each cell line was exposed to the test compound at a number of concentrations in triplicate for 48 h, with cisplatin and paclitaxel (Sigma) as positive controls. After incubation, MTS (100 *μ*g) was added to each well and the incubation continued for 4 h at 37 °C. After removal of 100 *μ*L of medium, the cells were lysed with 100 *μ*L of a solution of 20% SDS with 50% DMF. The optical density of the lysate was measured at 595 nm with a 96-well microtiter plate reader (Bio-Rad 680). The IC_50_ values of each compound were calculated by the Reed and Muench method.

### MTT assay

Cells were seeded into 96-well culture dishes at a density of 1 × 10^5^ cells/well in 100 *μ*L medium. After being cultured for 24 h, cells were treated with compounds in concentrations ranging from 2 to 64 *μ*M (using DMSO as the vehicle at a maximum concentration of 0.1%). Cells were incubated with various concentrations of the agents for 24 h, then the growth medium was changed and replaced with 200 *μ*L fresh medium with 10% FBS and 5 mg/mL MTT. Cells were incubated for 4 h and the medium was again replaced, this time with 200 *μ*L of DMSO. The absorbance of each well was measured at 570 nm using a microtiter plate reader. Experiments were conducted in triplicate. The results are presented as percentages of cell viability.

### Colony formation and sphere formation assays

For the colony formation assay, the P6C cells were seeded into a 6-well plate at a concentration of 100 cells per well. Then, the test compounds were added at different concentrations. After two weeks, the colonies were observed under a phase contrast microscope and stained with Coomassie Brilliant Blue R250. Each sample was analysed in triplicate, and this experiment was performed three times.

For the sphere formation assay, 1.5 mL of 1.2% agar in DMEM supplemented with 10% FBS was layered into each well of 6-well culture plates. Approximately 200 P6C cells were transplanted to the top of the 1.2% agar layer. To analyse the inhibitory effect of the compound on P6C cell anchorage-independent growth, the 1.2% agar layer and DEME were supplemented with the compound. Cells were incubated at 37 °C in 5% CO_2_ for three weeks, and the number of spheres was scored by Coomassie Brilliant Blue R250 staining.

### Physic-chemical Characters of the New Compounds

Bisebracteolasin A (**1**): colourless crystals from MeOH; mp 257–260 °C; [*α*] = −23.0 (*c* 0.2, MeOH); UV (MeOH) *λ*_max_ (log*ε*) 221.6 (4.33) nm; IR (KBr) *ν*_max_ 3425, 2927, 2861, 1764, 1742, 1704, 1680, 1633, 1462, 1409, 1384, 1325, 1309, 1251, 1222, 1183, 1080, 1041, 997, 972 cm^−1^; positive ESIMS *m/z* 665 [M + H]^+^; HREIMS *m/z* 687.3871 [M + Na]^+^ (calcd for C_40_H_56_O_8_Na, 687.3867); ^1^H and ^13^C NMR data, see Table 1.

Bisebracteolasin B (**2**): amorphous white powder; [*α*] = −30.1 (*c* 0.2 MeOH); UV (MeOH) *λ*_max_ (log*ε*) 215 (4.34) nm; IR (KBr) *ν*_max_ 3428, 2927, 2866, 1769, 1737, 1618, 1467, 1456, 1392, 1370, 1326, 1228, 1205, 1188, 1173, 1142, 1097, 1042, 960, 972 cm^−1^; positive ESIMS *m/z* 689 [M + Na]^+^; HRESIMS *m/z* 689.4019 [M + Na]^+^ (calcd for C_40_H_58_O_8_Na, 689.4024); ^1^H and ^13^C NMR data, see Table 1.

## Electronic supplementary material

Supplementary information
