# Supplementary material for: Two novel diterpenoid heterodimers, Bisebracteolasins A and B, from Euphorbia ebracteolata Hayata, and the cancer chemotherapeutic potential of Bisebracteolasin A
Source: Sci Rep. 2017 Nov 6;7:14507. doi: 10.1038/s41598-017-14637-w (PMC5674023; doi:10.1038/s41598-017-14637-w)
Supplement: Supplementary file 1 — Supplementary information [file 41598_2017_14637_MOESM1_ESM.pdf]

Supplementary Information

Two novel diterpenoid heterodimers, Bisebracteolasins A and B, from *Euphorbia ebracteolata* Hayata, and the cancer chemotherapeutic potential of Bisebracteolasin A

Wen-Juan Yuan[1,2,3,Δ], Xiao Ding[1,Δ], Zhe Wang[1], Bi-Juan Yang[1], Xiao-Nian Li[1], Yu Zhang[1], Duo-Zhi Chen[1], Shun-Lin Li[1], Quan Chen[4], Ying-Tong Di[1,]*, Haji Akber Aisa[2,]*, Xiao-Jiang Hao[1,]*

[1] State Key Laboratory of Phytochemistry and Plant Resources in West China, Kunming Institute of Botany, Chinese Academy of Sciences, Kunming 650204, P. R. China

[2] Xinjiang Technical Institute of Physics and Chemistry, Chinese Academy of Sciences, Urumqi 830011, Xinjiang, P. R. China

[3] University of Chinese Academy of Sciences, Beijing, 100049, P. R. China

[4] State Key Laboratory of Molecular Oncology, Cancer Institute/Hospital, Peking Union Medical College and Chinese Academy of Medical Sciences, Beijing, 100021, P. R. China.

No.	Contents	Page
1	Crystallographic data of Bisebracteolasin A **(1)**	2-15
2	**Figure S1.** MS/MS spectrum of Bisebracteolasin A (**1**)	16
3	**Figure S2.** MS/MS spectrum of Bisebracteolasin B (**2**)	17
4	**Figure S3** ^{1}H NMR spectrum of Bisebracteolasin A (**1**) in $CDCl_3$-d_1	18
5	**Figure S4** ^{13}C NMR spectrum of Bisebracteolasin A (**1**) in $CDCl_3$-d_1	18
6	**Figure S5** COSY spectrum of Bisebracteolasin A (**1**) in $CDCl_3$-d_1	19
7	**Figure S6** HSQC spectrum of Bisebracteolasin A (**1**) in $CDCl_3$-d_1	19
8	**Figure S7** HMBC spectrum of Bisebracteolasin A (**1**) in $CDCl_3$-d_1	20
9	**Figure S8** ROESY spectrum of Bisebracteolasin A (**1**) in $CDCl_3$-d_1	20
10	**Figure S9** HRESIMS spectrums of Bisebracteolasin A (**1**)	21
11	**Figure S10** UV spectrum of Bisebracteolasin A (**1**) in Methanol	22
12	**Figure S11** IR spectrum of Bisebracteolasin A (**1**)	23
13	**Figure S12** CD spectrum of Bisebracteolasin A (**1**)	24-25
14	**Figure S13** ^{1}H NMR spectrum of Bisebracteolasin B (**2**) in $CDCl_3$-d_1	26
15	**Figure S14** ^{13}C NMR spectrum of Bisebracteolasin B (**2**) in $CDCl_3$-d_1	26
16	**Figure S15** COSY spectrum of Bisebracteolasin B (**2**) in $CDCl_3$-d_1	27
17	**Figure S16** HSQC spectrum of Bisebracteolasin B (**2**) in $CDCl_3$-d_1	27
18	**Figure S17** HMBC spectrum of Bisebracteolasin B (**2**) in $CDCl_3$-d_1	28
19	**Figure S18** ROESY spectrum of Bisebracteolasin B (**2**) in $CDCl_3$-d_1	28
20	**Figure S19** HRESIMS spectrums of Bisebracteolasin B (**2**)	29
21	**Figure S20** UV spectrum of Bisebracteolasin B (**2**) in Methanol	30
22	**Figure S21** IR spectrum of Bisebracteolasin B (**2**)	31
23	**Figure S22** CD spectrum of Bisebracteolasin B (**2**)	32-33

Crystallographic data of Bisebracteolasin A (1)

Crystals of 1 were used for measurement on a Bruker APEX DUO diffractometer with gphirate monochromater Cu Kα radiation. Crystal structures were solved by direct methods with SHELXS-2014, expanded using difference Fourier technique, and refined with full-matrix least-squares on F2 using SHELXS-2014. Non-hydrogen atoms were refined anisotropically. Hydrogen atom were placed in idealized positions and refined using a riding model. Crystallographic data for compounds **1** has been deposited in the Cambridge Crystallographic Data Centre (deposition numbers: CCDC 1521406). Copies of these data can be obtained free of charge via www.ccdc.cam.ac.uk (or from the Cambridge Crystallographic Data Centre, 12, Union Road, CAMBRIDGE CB2 1EZ, UK.; fax: (+44) 1223-336-033; or deposit@ccdc.cam.ac.uk).

Crystal data for cu_hyx51_0m: $C_{40}H_{56}O_8$, M = 664.85, orthorhombic, a = 11.1592(4) Å, b = 13.2190(5) Å, c = 23.8712(9) Å, α = 90.00 °, β = 90.00 °, γ = 90.00 °, V = 3521.3(2) Å^3, T = 100(2) K, space group P212121, Z = 4, μ(CuKα) = 0.689 mm^{-1}, 16311 reflections measured, 5825 independent reflections (R_{int} = 0.0636). The final R_1 values were 0.0623 ($I > 2\sigma(I)$). The final $wR(F^2)$ values were 0.1730 ($I > 2\sigma(I)$). The final R_1 values were 0.0813 (all data). The final $wR(F^2)$ values were 0.2186 (all data). The goodness of fit on F^2 was 1.146. Flack parameter = 0.1(3). The Hooft parameter is 0.11(10) for 2372 Bijvoet pairs.

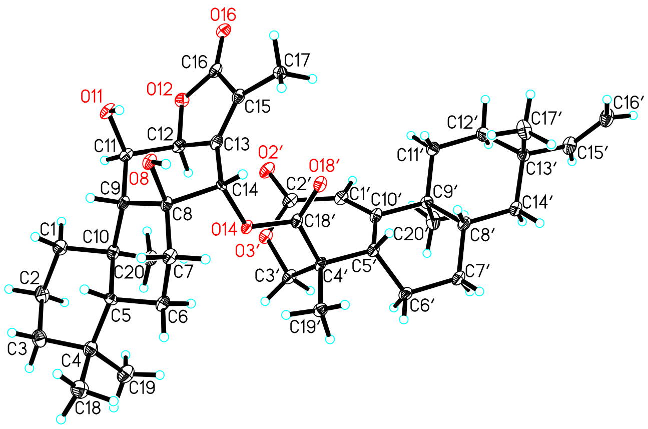

View of a molecule of hyx51 with the atom-labelling scheme.

Displacement ellipsoids are drawn at the 30% probability level.

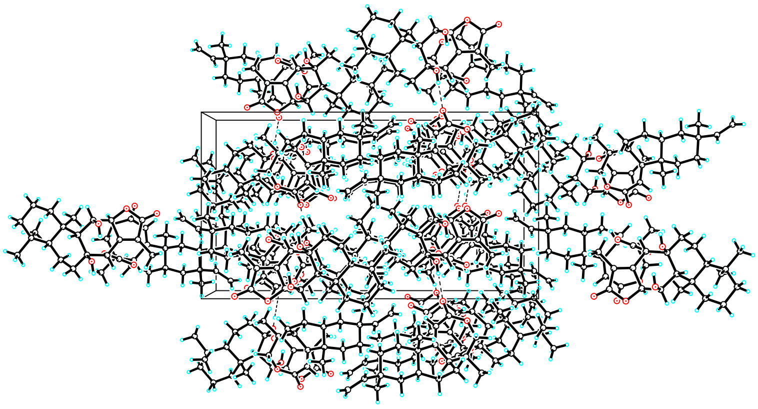

View of the hydrogen-bonded motif of hyx51.

Hydrogen-bonds are shown as dashed lines.

Table 1. Crystal data and structure refinement for hyx51.

Identification code	cu_hyx51_0m
Empirical formula	C40 H56 O8
Formula weight	664.85
Temperature	100(2) K
Wavelength	1.54178 A
Crystal system, space group	Orthorhombic, P 21 21 21
Unit cell dimensions	a = 11.1592(4) A alpha = 90 deg.
	b = 13.2190(5) A beta = 90 deg.
	c = 23.8712(9) A gamma = 90 deg.
Volume	3521.3(2) A^3
Z, Calculated density	4, 1.254 Mg/m^3
Absorption coefficient	0.689 mm^-1
F(000)	1440

Crystal size	0.33 x 0.11 x 0.09 mm
Theta range for data collection	3.70 to 69.40 deg.
Limiting indices	-12<=h<=13, -15<=k<=14, -27<=l<=27
Reflections collected / unique	16311 / 5825 [R(int) = 0.0636]
Completeness to theta = 69.40	93.2 %
Absorption correction	Semi-empirical from equivalents
Max. and min. transmission	0.9406 and 0.8046
Refinement method	Full-matrix least-squares on F^2
Data / restraints / parameters	5825 / 0 / 443
Goodness-of-fit on F^2	1.146
Final R indices [I>2sigma(I)]	R1 = 0.0623, wR2 = 0.1730
R indices (all data)	R1 = 0.0813, wR2 = 0.2186
Absolute structure parameter	0.1(3)
Extinction coefficient	0.0009(3)
Largest diff. peak and hole	0.476 and -0.510 e.A^-3

Table 2. Atomic coordinates (x 10^4) and equivalent isotropic displacement parameters (A^2 x 10^3) for hyx51.U(eq) is defined as one third of the trace of the orthogonalized Uij tensor.

	x	y	z	U(eq)
O(14)	988(2)	2604(2)	7949(1)	19(1)
O(12)	-1040(3)	171(2)	7146(1)	24(1)
O(16)	-1409(2)	428(2)	6234(1)	27(1)
O(11)	-2812(2)	960(2)	7833(1)	28(1)
O(18')	1483(2)	3227(2)	7100(1)	24(1)
O(3')	2612(2)	1027(2)	7810(1)	23(1)
O(2')	2147(3)	28(2)	7119(1)	30(1)
O(8)	-2233(2)	2941(2)	8075(1)	21(1)

C(3)	-643(4)	869(3)	10302(2)	31(1)
C(4)	103(4)	1742(3)	10052(2)	27(1)
C(5)	-417(3)	2020(3)	9474(2)	20(1)
C(10)	-597(3)	1137(3)	9043(2)	22(1)
C(9)	-1388(3)	1553(2)	8544(2)	20(1)
C(8)	-1082(3)	2623(2)	8301(2)	18(1)
C(14)	-260(3)	2667(3)	7772(1)	15(1)
C(18')	1776(3)	2920(2)	7560(2)	18(1)
C(4')	3067(3)	2866(3)	7764(2)	19(1)
C(5')	3925(3)	3054(2)	7263(2)	20(1)
C(10')	4056(3)	2182(3)	6860(2)	21(1)
C(9')	4897(3)	2401(3)	6355(2)	20(1)
C(8')	5290(4)	3521(3)	6373(2)	26(1)
C(14')	6053(4)	3816(3)	5862(2)	28(1)
C(13')	5434(3)	3635(3)	5302(2)	23(1)
C(15')	6359(4)	3725(3)	4844(2)	30(1)
C(16')	6307(4)	4279(3)	4382(2)	35(1)
C(18)	-48(5)	2657(3)	10447(2)	36(1)
C(6)	192(3)	2919(3)	9183(2)	23(1)
C(7)	-659(3)	3369(3)	8745(2)	22(1)
C(13)	-545(3)	1828(3)	7383(2)	21(1)
C(12)	-701(3)	792(3)	7620(2)	22(1)
C(16)	-1121(3)	772(3)	6679(2)	23(1)
C(15)	-798(3)	1817(3)	6827(2)	22(1)
C(17)	-773(3)	2641(3)	6402(2)	23(1)
C(11)	-1663(3)	777(3)	8075(2)	21(1)
C(1)	-1349(4)	318(3)	9346(2)	27(1)
C(2)	-805(4)	-13(3)	9902(2)	30(1)
C(20)	576(3)	667(3)	8833(2)	26(1)
C(3')	3304(3)	1842(3)	8049(2)	25(1)
C(2')	2753(4)	752(3)	7275(2)	25(1)
C(1')	3589(3)	1249(3)	6889(2)	23(1)
C(11')	4216(4)	2244(3)	5808(2)	26(1)
C(12')	4967(4)	2537(3)	5297(2)	28(1)
C(7')	5897(4)	3772(4)	6925(2)	39(1)
C(6')	5192(3)	3397(3)	7436(2)	24(1)
C(17')	4400(4)	4370(3)	5204(2)	35(1)
C(20')	5969(4)	1676(3)	6394(2)	36(1)
C(19')	3204(3)	3699(3)	8210(2)	23(1)
C(19)	1439(4)	1468(3)	10064(2)	38(1)

Table 3. Bond lengths [A] and angles [deg] for hyx51.

O(14)-C(18')	1.345(5)
O(14)-C(14)	1.458(4)
O(12)-C(16)	1.372(5)
O(12)-C(12)	1.448(5)
O(16)-C(16)	1.200(5)
O(11)-C(11)	1.427(4)
O(11)-H(11)	0.8400
O(18')-C(18')	1.216(5)
O(3')-C(2')	1.337(5)
O(3')-C(3')	1.444(4)
O(2')-C(2')	1.230(5)
O(8)-C(8)	1.455(4)
O(8)-H(8)	0.8400
C(3)-C(2)	1.517(6)
C(3)-C(4)	1.542(6)
C(3)-H(3A)	0.9900
C(3)-H(3B)	0.9900
C(4)-C(19)	1.534(6)
C(4)-C(18)	1.543(6)
C(4)-C(5)	1.543(5)
C(5)-C(6)	1.535(5)
C(5)-C(10)	1.569(5)
C(5)-H(5)	1.0000
C(10)-C(20)	1.533(5)
C(10)-C(1)	1.549(5)
C(10)-C(9)	1.582(5)
C(9)-C(11)	1.549(5)
C(9)-C(8)	1.566(4)
C(9)-H(9)	1.0000
C(8)-C(7)	1.522(5)
C(8)-C(14)	1.562(5)
C(14)-C(13)	1.480(5)
C(14)-H(14)	1.0000
C(18')-C(4')	1.522(5)
C(4')-C(3')	1.537(5)
C(4')-C(19')	1.540(5)
C(4')-C(5')	1.552(5)
C(5')-C(10')	1.508(5)
C(5')-C(6')	1.542(5)
C(5')-H(5')	1.0000
C(10')-C(1')	1.341(5)

C(10')-C(9')	1.556(5)
C(9')-C(11')	1.524(6)
C(9')-C(20')	1.536(5)
C(9')-C(8')	1.544(5)
C(8')-C(7')	1.518(6)
C(8')-C(14')	1.538(6)
C(8')-H(8')	1.0000
C(14')-C(13')	1.526(6)
C(14')-H(14A)	0.9900
C(14')-H(14B)	0.9900
C(13')-C(15')	1.507(6)
C(13')-C(17')	1.526(6)
C(13')-C(12')	1.542(5)
C(15')-C(16')	1.326(6)
C(15')-H(15')	0.9500
C(16')-H(16A)	0.9500
C(16')-H(16B)	0.9500
C(18)-H(18A)	0.9800
C(18)-H(18B)	0.9800
C(18)-H(18C)	0.9800
C(6)-C(7)	1.532(5)
C(6)-H(6A)	0.9900
C(6)-H(6B)	0.9900
C(7)-H(7A)	0.9900
C(7)-H(7B)	0.9900
C(13)-C(15)	1.357(6)
C(13)-C(12)	1.492(5)
C(12)-C(11)	1.527(6)
C(12)-H(12)	1.0000
C(16)-C(15)	1.471(5)
C(15)-C(17)	1.490(5)
C(17)-H(17A)	0.9800
C(17)-H(17B)	0.9800
C(17)-H(17C)	0.9800
C(11)-H(11A)	1.0000
C(1)-C(2)	1.525(6)
C(1)-H(1A)	0.9900
C(1)-H(1B)	0.9900
C(2)-H(2A)	0.9900
C(2)-H(2B)	0.9900
C(20)-H(20A)	0.9800
C(20)-H(20B)	0.9800
C(20)-H(20C)	0.9800
C(3')-H(3'1)	0.9900

C(3')-H(3'2)	0.9900
C(2')-C(1')	1.467(6)
C(1')-H(1')	0.9500
C(11')-C(12')	1.531(6)
C(11')-H(11B)	0.9900
C(11')-H(11C)	0.9900
C(12')-H(12A)	0.9900
C(12')-H(12B)	0.9900
C(7')-C(6')	1.534(6)
C(7')-H(7'1)	0.9900
C(7')-H(7'2)	0.9900
C(6')-H(6'1)	0.9900
C(6')-H(6'2)	0.9900
C(17')-H(17D)	0.9800
C(17')-H(17E)	0.9800
C(17')-H(17F)	0.9800
C(20')-H(20D)	0.9800
C(20')-H(20E)	0.9800
C(20')-H(20F)	0.9800
C(19')-H(19A)	0.9800
C(19')-H(19B)	0.9800
C(19')-H(19C)	0.9800
C(19)-H(19D)	0.9800
C(19)-H(19E)	0.9800
C(19)-H(19F)	0.9800
C(18')-O(14)-C(14)	114.0(3)
C(16)-O(12)-C(12)	108.9(3)
C(11)-O(11)-H(11)	109.5
C(2')-O(3')-C(3')	121.1(3)
C(8)-O(8)-H(8)	109.5
C(2)-C(3)-C(4)	113.5(4)
C(2)-C(3)-H(3A)	108.9
C(4)-C(3)-H(3A)	108.9
C(2)-C(3)-H(3B)	108.9
C(4)-C(3)-H(3B)	108.9
H(3A)-C(3)-H(3B)	107.7
C(19)-C(4)-C(3)	110.0(3)
C(19)-C(4)-C(18)	106.3(4)
C(3)-C(4)-C(18)	107.0(4)
C(19)-C(4)-C(5)	116.0(4)
C(3)-C(4)-C(5)	108.7(3)
C(18)-C(4)-C(5)	108.6(3)
C(6)-C(5)-C(4)	115.0(3)

C(6)-C(5)-C(10)	109.7(3)
C(4)-C(5)-C(10)	117.2(3)
C(6)-C(5)-H(5)	104.5
C(4)-C(5)-H(5)	104.5
C(10)-C(5)-H(5)	104.5
C(20)-C(10)-C(1)	109.4(3)
C(20)-C(10)-C(5)	114.0(3)
C(1)-C(10)-C(5)	106.4(3)
C(20)-C(10)-C(9)	111.8(3)
C(1)-C(10)-C(9)	107.0(3)
C(5)-C(10)-C(9)	107.8(3)
C(11)-C(9)-C(8)	112.0(3)
C(11)-C(9)-C(10)	115.1(3)
C(8)-C(9)-C(10)	118.1(3)
C(11)-C(9)-H(9)	103.0
C(8)-C(9)-H(9)	103.0
C(10)-C(9)-H(9)	103.0
O(8)-C(8)-C(7)	110.2(3)
O(8)-C(8)-C(14)	102.0(3)
C(7)-C(8)-C(14)	110.9(3)
O(8)-C(8)-C(9)	101.9(3)
C(7)-C(8)-C(9)	113.3(3)
C(14)-C(8)-C(9)	117.4(3)
O(14)-C(14)-C(13)	110.1(3)
O(14)-C(14)-C(8)	108.9(3)
C(13)-C(14)-C(8)	110.7(3)
O(14)-C(14)-H(14)	109.0
C(13)-C(14)-H(14)	109.0
C(8)-C(14)-H(14)	109.0
O(18')-C(18')-O(14)	123.4(3)
O(18')-C(18')-C(4')	124.0(3)
O(14)-C(18')-C(4')	112.6(3)
C(18')-C(4')-C(3')	110.2(3)
C(18')-C(4')-C(19')	106.4(3)
C(3')-C(4')-C(19')	107.8(3)
C(18')-C(4')-C(5')	109.3(3)
C(3')-C(4')-C(5')	112.1(3)
C(19')-C(4')-C(5')	110.9(3)
C(10')-C(5')-C(6')	107.9(3)
C(10')-C(5')-C(4')	115.4(3)
C(6')-C(5')-C(4')	114.0(3)
C(10')-C(5')-H(5')	106.3
C(6')-C(5')-H(5')	106.3
C(4')-C(5')-H(5')	106.3

C(1')-C(10')-C(5')	129.3(4)
C(1')-C(10')-C(9')	116.5(3)
C(5')-C(10')-C(9')	114.2(3)
C(11')-C(9')-C(20')	110.8(3)
C(11')-C(9')-C(8')	107.3(3)
C(20')-C(9')-C(8')	112.1(3)
C(11')-C(9')-C(10')	109.7(3)
C(20')-C(9')-C(10')	107.9(3)
C(8')-C(9')-C(10')	109.1(3)
C(7')-C(8')-C(14')	112.6(3)
C(7')-C(8')-C(9')	111.1(4)
C(14')-C(8')-C(9')	112.2(3)
C(7')-C(8')-H(8')	106.8
C(14')-C(8')-H(8')	106.8
C(9')-C(8')-H(8')	106.8
C(13')-C(14')-C(8')	113.9(3)
C(13')-C(14')-H(14A)	108.8
C(8')-C(14')-H(14A)	108.8
C(13')-C(14')-H(14B)	108.8
C(8')-C(14')-H(14B)	108.8
H(14A)-C(14')-H(14B)	107.7
C(15')-C(13')-C(14')	108.2(3)
C(15')-C(13')-C(17')	110.9(3)
C(14')-C(13')-C(17')	112.1(4)
C(15')-C(13')-C(12')	107.5(3)
C(14')-C(13')-C(12')	107.9(3)
C(17')-C(13')-C(12')	110.1(4)
C(16')-C(15')-C(13')	128.1(4)
C(16')-C(15')-H(15')	116.0
C(13')-C(15')-H(15')	116.0
C(15')-C(16')-H(16A)	120.0
C(15')-C(16')-H(16B)	120.0
H(16A)-C(16')-H(16B)	120.0
C(4)-C(18)-H(18A)	109.5
C(4)-C(18)-H(18B)	109.5
H(18A)-C(18)-H(18B)	109.5
C(4)-C(18)-H(18C)	109.5
H(18A)-C(18)-H(18C)	109.5
H(18B)-C(18)-H(18C)	109.5
C(7)-C(6)-C(5)	109.5(3)
C(7)-C(6)-H(6A)	109.8
C(5)-C(6)-H(6A)	109.8
C(7)-C(6)-H(6B)	109.8
C(5)-C(6)-H(6B)	109.8

H(6A)-C(6)-H(6B)	108.2
C(8)-C(7)-C(6)	114.6(3)
C(8)-C(7)-H(7A)	108.6
C(6)-C(7)-H(7A)	108.6
C(8)-C(7)-H(7B)	108.6
C(6)-C(7)-H(7B)	108.6
H(7A)-C(7)-H(7B)	107.6
C(15)-C(13)-C(14)	131.8(3)
C(15)-C(13)-C(12)	109.7(3)
C(14)-C(13)-C(12)	118.3(3)
O(12)-C(12)-C(13)	104.8(3)
O(12)-C(12)-C(11)	111.3(3)
C(13)-C(12)-C(11)	111.4(3)
O(12)-C(12)-H(12)	109.8
C(13)-C(12)-H(12)	109.8
C(11)-C(12)-H(12)	109.8
O(16)-C(16)-O(12)	121.2(3)
O(16)-C(16)-C(15)	129.4(4)
O(12)-C(16)-C(15)	109.4(3)
C(13)-C(15)-C(16)	107.2(3)
C(13)-C(15)-C(17)	130.9(4)
C(16)-C(15)-C(17)	121.9(4)
C(15)-C(17)-H(17A)	109.5
C(15)-C(17)-H(17B)	109.5
H(17A)-C(17)-H(17B)	109.5
C(15)-C(17)-H(17C)	109.5
H(17A)-C(17)-H(17C)	109.5
H(17B)-C(17)-H(17C)	109.5
O(11)-C(11)-C(12)	110.0(3)
O(11)-C(11)-C(9)	111.0(3)
C(12)-C(11)-C(9)	111.4(3)
O(11)-C(11)-H(11A)	108.1
C(12)-C(11)-H(11A)	108.1
C(9)-C(11)-H(11A)	108.1
C(2)-C(1)-C(10)	113.1(3)
C(2)-C(1)-H(1A)	109.0
C(10)-C(1)-H(1A)	109.0
C(2)-C(1)-H(1B)	109.0
C(10)-C(1)-H(1B)	109.0
H(1A)-C(1)-H(1B)	107.8
C(3)-C(2)-C(1)	111.9(3)
C(3)-C(2)-H(2A)	109.2
C(1)-C(2)-H(2A)	109.2
C(3)-C(2)-H(2B)	109.2

C(1)-C(2)-H(2B)	109.2
H(2A)-C(2)-H(2B)	107.9
C(10)-C(20)-H(20A)	109.5
C(10)-C(20)-H(20B)	109.5
H(20A)-C(20)-H(20B)	109.5
C(10)-C(20)-H(20C)	109.5
H(20A)-C(20)-H(20C)	109.5
H(20B)-C(20)-H(20C)	109.5
O(3')-C(3')-C(4')	113.0(3)
O(3')-C(3')-H(3'1)	109.0
C(4')-C(3')-H(3'1)	109.0
O(3')-C(3')-H(3'2)	109.0
C(4')-C(3')-H(3'2)	109.0
H(3'1)-C(3')-H(3'2)	107.8
O(2')-C(2')-O(3')	115.8(4)
O(2')-C(2')-C(1')	120.5(4)
O(3')-C(2')-C(1')	123.7(3)
C(10')-C(1')-C(2')	133.7(4)
C(10')-C(1')-H(1')	113.1
C(2')-C(1')-H(1')	113.1
C(9')-C(11')-C(12')	112.0(3)
C(9')-C(11')-H(11B)	109.2
C(12')-C(11')-H(11B)	109.2
C(9')-C(11')-H(11C)	109.2
C(12')-C(11')-H(11C)	109.2
H(11B)-C(11')-H(11C)	107.9
C(11')-C(12')-C(13')	114.7(3)
C(11')-C(12')-H(12A)	108.6
C(13')-C(12')-H(12A)	108.6
C(11')-C(12')-H(12B)	108.6
C(13')-C(12')-H(12B)	108.6
H(12A)-C(12')-H(12B)	107.6
C(8')-C(7')-C(6')	113.0(3)
C(8')-C(7')-H(7'1)	109.0
C(6')-C(7')-H(7'1)	109.0
C(8')-C(7')-H(7'2)	109.0
C(6')-C(7')-H(7'2)	109.0
H(7'1)-C(7')-H(7'2)	107.8
C(7')-C(6')-C(5')	110.6(3)
C(7')-C(6')-H(6'1)	109.5
C(5')-C(6')-H(6'1)	109.5
C(7')-C(6')-H(6'2)	109.5
C(5')-C(6')-H(6'2)	109.5
H(6'1)-C(6')-H(6'2)	108.1

C(13')-C(17')-H(17D)	109.5
C(13')-C(17')-H(17E)	109.5
H(17D)-C(17')-H(17E)	109.5
C(13')-C(17')-H(17F)	109.5
H(17D)-C(17')-H(17F)	109.5
H(17E)-C(17')-H(17F)	109.5
C(9')-C(20')-H(20D)	109.5
C(9')-C(20')-H(20E)	109.5
H(20D)-C(20')-H(20E)	109.5
C(9')-C(20')-H(20F)	109.5
H(20D)-C(20')-H(20F)	109.5
H(20E)-C(20')-H(20F)	109.5
C(4')-C(19')-H(19A)	109.5
C(4')-C(19')-H(19B)	109.5
H(19A)-C(19')-H(19B)	109.5
C(4')-C(19')-H(19C)	109.5
H(19A)-C(19')-H(19C)	109.5
H(19B)-C(19')-H(19C)	109.5
C(4)-C(19)-H(19D)	109.5
C(4)-C(19)-H(19E)	109.5
H(19D)-C(19)-H(19E)	109.5
C(4)-C(19)-H(19F)	109.5
H(19D)-C(19)-H(19F)	109.5
H(19E)-C(19)-H(19F)	109.5

Symmetry transformations used to generate equivalent atoms:

Table 4. Anisotropic displacement parameters (A^2 x 10^3) for hyx51. The anisotropic displacement factor exponent takes the form: -2 pi^2 [h^2 a*^2 U11 + ... + 2 h k a* b* U12]

	U11	U22	U33	U23	U13	U12
O(14)	13(1)	19(1)	26(2)	5(1)	-1(1)	-1(1)
O(12)	27(1)	19(1)	27(2)	-4(1)	1(1)	-2(1)
O(16)	23(1)	26(1)	31(2)	-3(1)	-2(1)	-4(1)
O(11)	15(1)	27(1)	42(2)	1(1)	-4(1)	-4(1)
O(18')	19(1)	21(1)	32(2)	4(1)	2(1)	0(1)
O(3')	22(1)	15(1)	32(2)	-1(1)	1(1)	1(1)
O(2')	28(2)	16(1)	47(2)	-2(1)	-2(1)	-4(1)
O(8)	15(1)	18(1)	31(2)	4(1)	-1(1)	2(1)
C(3)	36(2)	27(2)	31(2)	3(2)	6(2)	0(2)
C(4)	25(2)	24(2)	31(2)	3(2)	0(2)	-1(2)
C(5)	24(2)	21(2)	14(2)	0(1)	-1(1)	0(1)
C(10)	20(2)	18(2)	29(2)	6(2)	2(2)	0(1)
C(9)	16(2)	17(2)	26(2)	5(1)	-1(2)	-2(1)
C(8)	13(2)	17(2)	25(2)	4(1)	2(2)	2(1)
C(14)	16(2)	19(2)	10(2)	3(1)	-1(1)	-2(1)
C(18')	18(2)	12(1)	25(2)	4(1)	1(2)	0(1)
C(4')	12(2)	18(2)	27(2)	-4(1)	5(1)	0(1)
C(5')	16(2)	18(2)	26(2)	2(1)	-1(1)	-1(1)
C(10')	10(2)	19(2)	33(2)	3(2)	2(1)	4(1)
C(9')	17(2)	21(2)	22(2)	-2(2)	1(2)	2(1)
C(8')	21(2)	24(2)	32(2)	2(2)	-2(2)	-6(1)
C(14')	23(2)	32(2)	30(2)	1(2)	3(2)	-8(2)
C(13')	22(2)	23(2)	25(2)	6(2)	0(2)	2(2)
C(15')	20(2)	27(2)	43(3)	3(2)	8(2)	6(2)
C(16')	24(2)	31(2)	49(3)	7(2)	5(2)	1(2)
C(18)	44(3)	28(2)	34(3)	5(2)	-2(2)	-3(2)
C(6)	20(2)	17(2)	32(2)	0(2)	1(2)	-2(1)
C(7)	21(2)	16(2)	30(2)	1(1)	0(2)	2(1)
C(13)	14(2)	19(2)	30(2)	-1(2)	3(2)	2(1)
C(12)	18(2)	18(2)	29(2)	-6(2)	0(2)	0(1)
C(16)	14(2)	22(2)	33(2)	-1(2)	1(2)	0(1)
C(15)	14(2)	22(2)	31(2)	2(2)	1(2)	2(1)
C(17)	24(2)	24(2)	22(2)	1(2)	-2(2)	-1(2)
C(11)	17(2)	18(2)	28(2)	1(1)	-4(2)	-2(1)
C(1)	32(2)	19(2)	30(2)	1(2)	0(2)	-3(2)
C(2)	37(2)	20(2)	32(3)	9(2)	4(2)	0(2)
C(20)	20(2)	24(2)	32(2)	1(2)	2(2)	4(2)

C(3')	20(2)	18(2)	36(2)	1(2)	-3(2)	0(1)
C(2')	22(2)	14(2)	39(2)	-2(2)	-4(2)	3(1)
C(1')	20(2)	17(2)	31(2)	-5(2)	2(2)	2(1)
C(11')	29(2)	22(2)	28(2)	0(2)	-2(2)	-5(2)
C(12')	34(2)	24(2)	28(2)	1(2)	-1(2)	-2(2)
C(7')	30(2)	55(3)	32(3)	-3(2)	2(2)	-24(2)
C(6')	15(2)	29(2)	28(2)	-1(2)	-2(2)	-1(2)
C(17')	30(2)	34(2)	41(3)	14(2)	9(2)	14(2)
C(20')	23(2)	38(2)	46(3)	13(2)	8(2)	13(2)
C(19')	20(2)	24(2)	27(2)	-4(2)	0(2)	0(2)
C(19)	28(2)	37(2)	48(3)	2(2)	-2(2)	0(2)

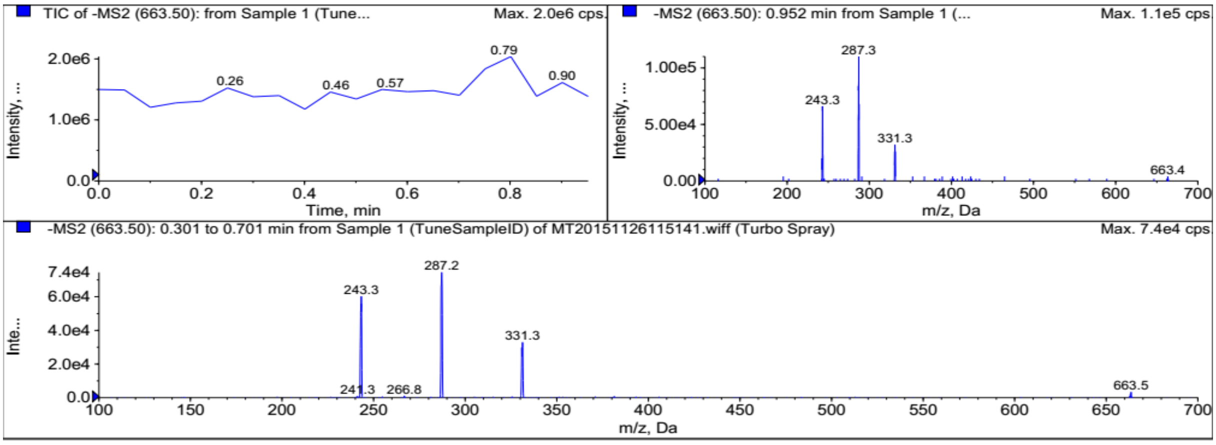

Figure S1. MS/MS spectrum of Bisebracteolasin A (1)

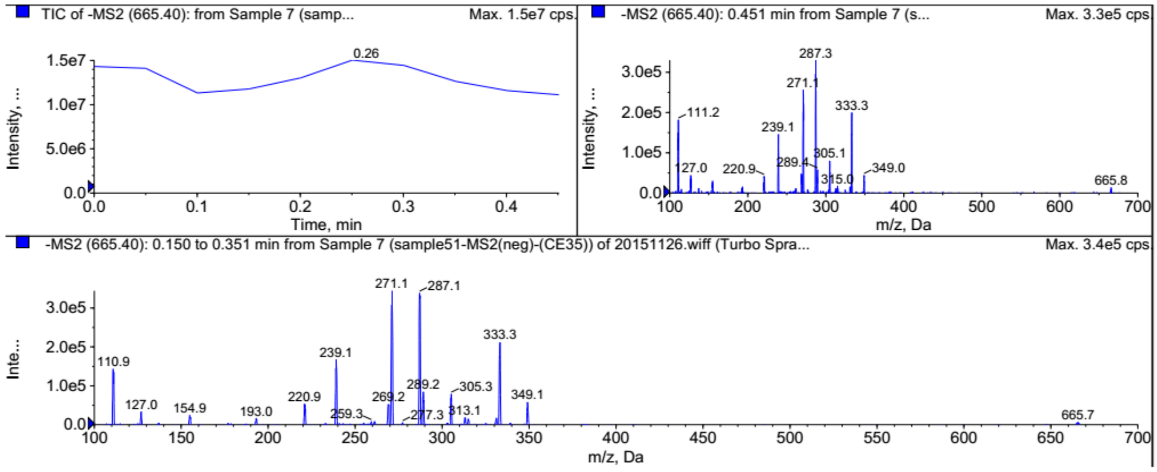

Figure S2. MS/MS spectrum of Bisebracteolasin B (2)

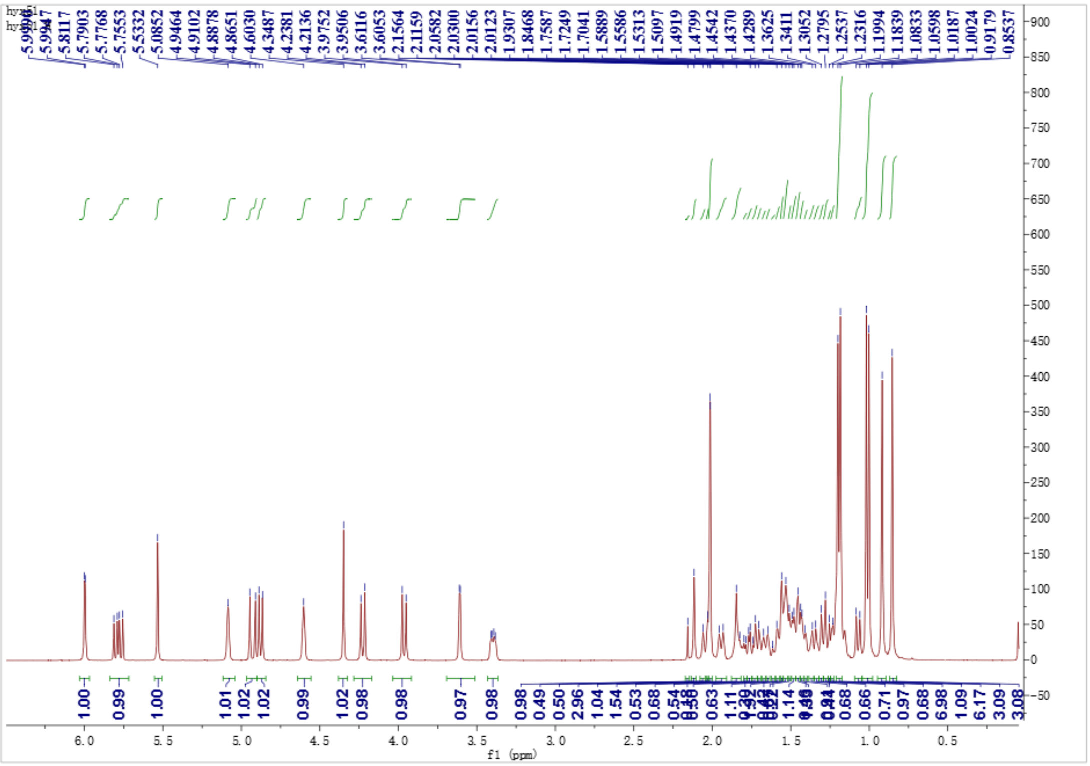

S3 ^{1}H NMR spectrum of Bisebracteolasin A (1) in $CDCl_3$-d_1

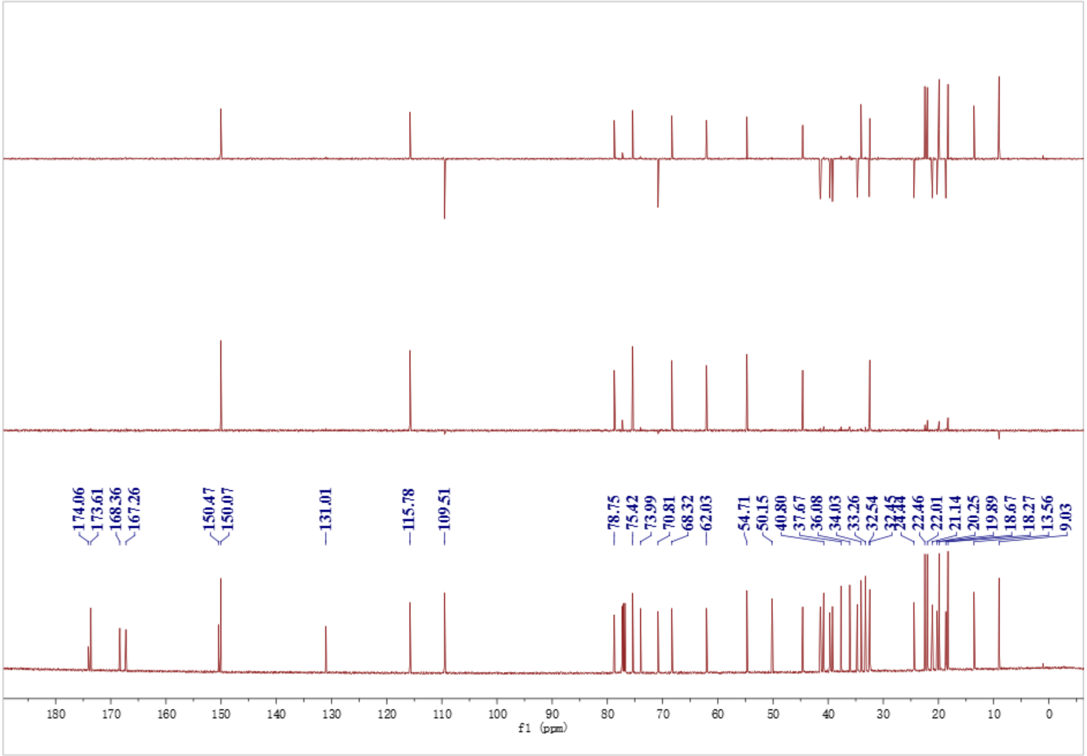

S4 ^{13}C NMR spectrum of Bisebracteolasin A (1) in $CDCl_3$-d_1

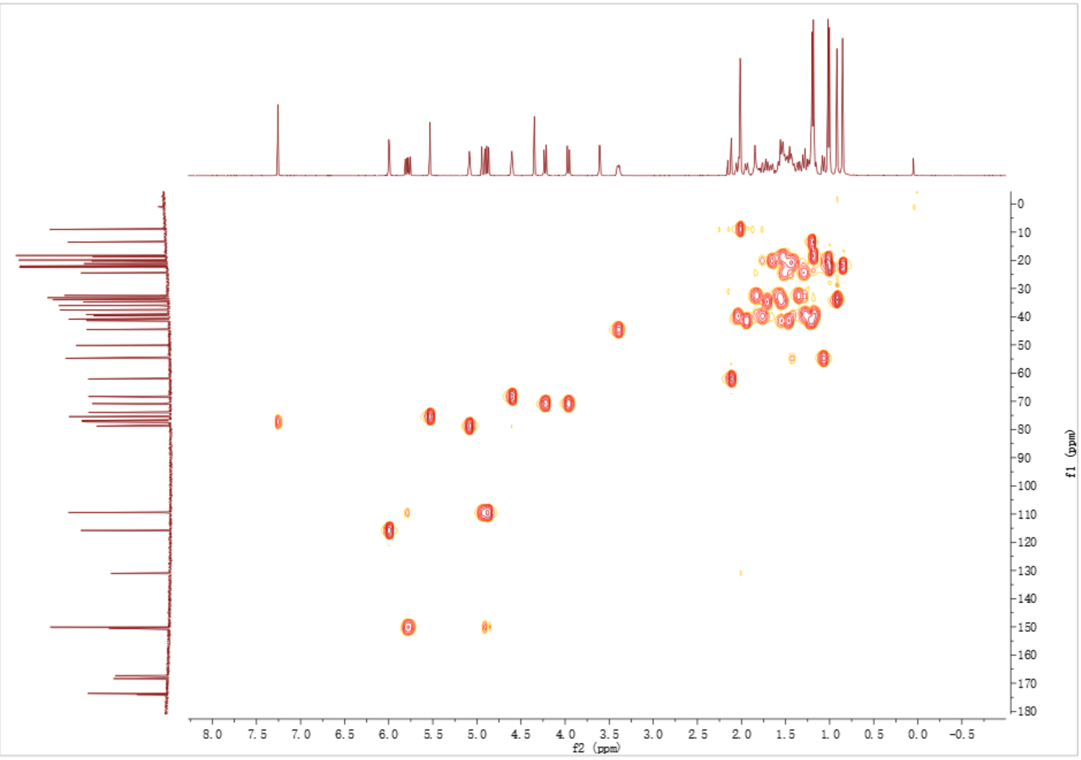

S5 HSQC spectrum of Bisebracteolasin A (1) in $CDCl_3$-d_1

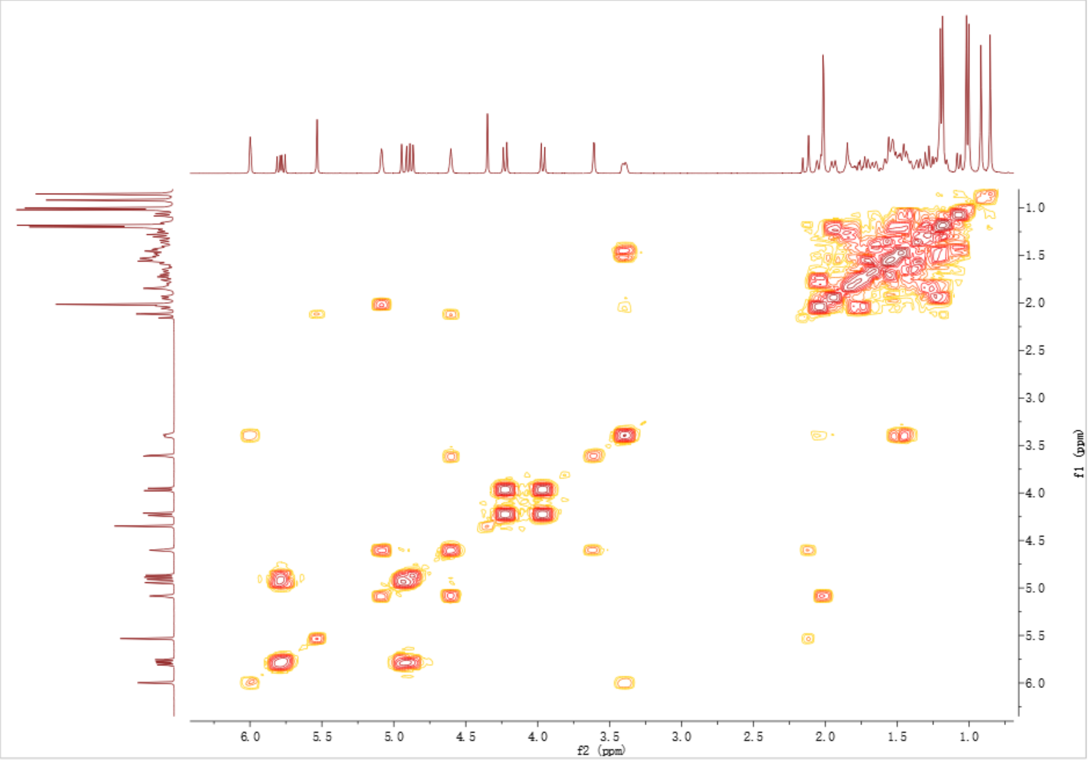

S6 COSY spectrum of Bisebracteolasin A (1) in $CDCl_3$-d_1

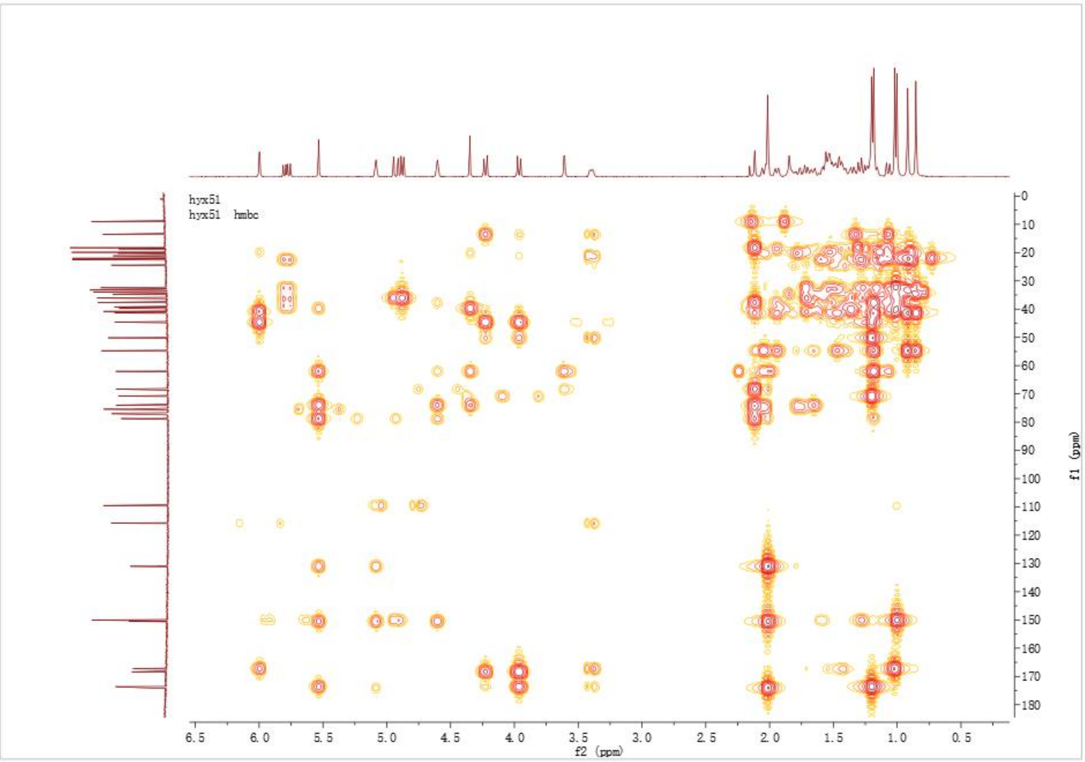

S7 HMBC spectrum of Bisebracteolasin A (1) in $CDCl_3$-d_1

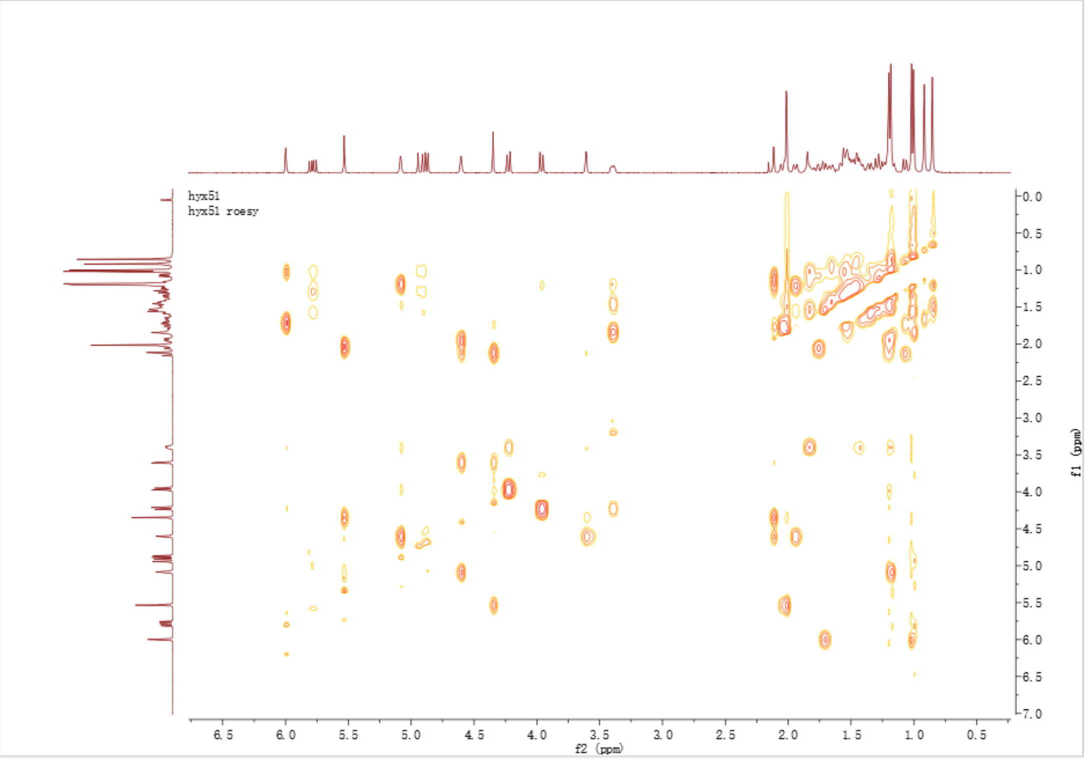

S8 ROESY spectrum of Bisebracteolasin A (1) in $CDCl_3$-d_1

Qualitative Analysis Report

**Data Filename**	hyx51.d	**Sample Name**	hyx51
**Sample Type**	Sample	**Position**	P1-B8
**Instrument Name**	Instrument 1	**User Name**	
**Acq Method**	SIBU.m	**Acquired Time**	10/14/2015 10:35:52 AM
**IRM Calibration Status**	[redacted]	**DA Method**	ESI+.m
**Comment**			

Sample Group **Info.**

Acquisition SW Version 6200 series TOF/6500 series Q-TOF B.05.01 (B5125.2)

User Spectra

Fragmentor Voltage	Collision Energy	Ionization Mode
135	0	ESI

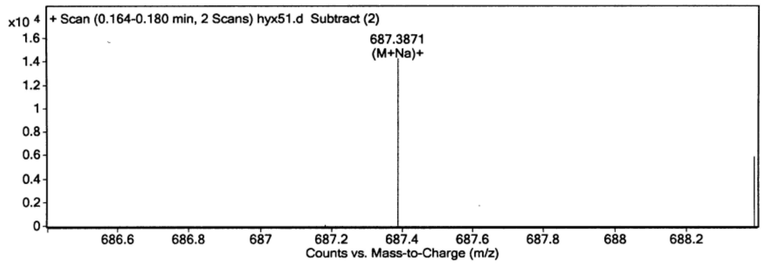

Peak List

m/z	z	Abund	Formula	Ion
385.2189	2	2914		
393.207	2	4518.94		
682.4315	1	19883.62		
683.4362	1	8703.2		
687.3871	1	14246.87	C40 H56 O8	(M+Na)+
688.3904	1	5899.63	C40 H56 O8	(M+Na)+
703.3615	1	14612.67		
704.3647	1	6210.6		

Formula Calculator Element Limits

Element	Min	Max
C	3	60
H	0	120
O	0	30

Formula Calculator Results

Formula	CalculatedMass	CalculatedMz	Mz	Diff. (mDa)	Diff. (ppm)	DBE
C40 H56 O8	664.3975	687.3867	687.3871	-0.3	-0.5	13.0000

--- End Of Report ---

S9 HRESIMS spectrums of Bisebracteolasin A (1)

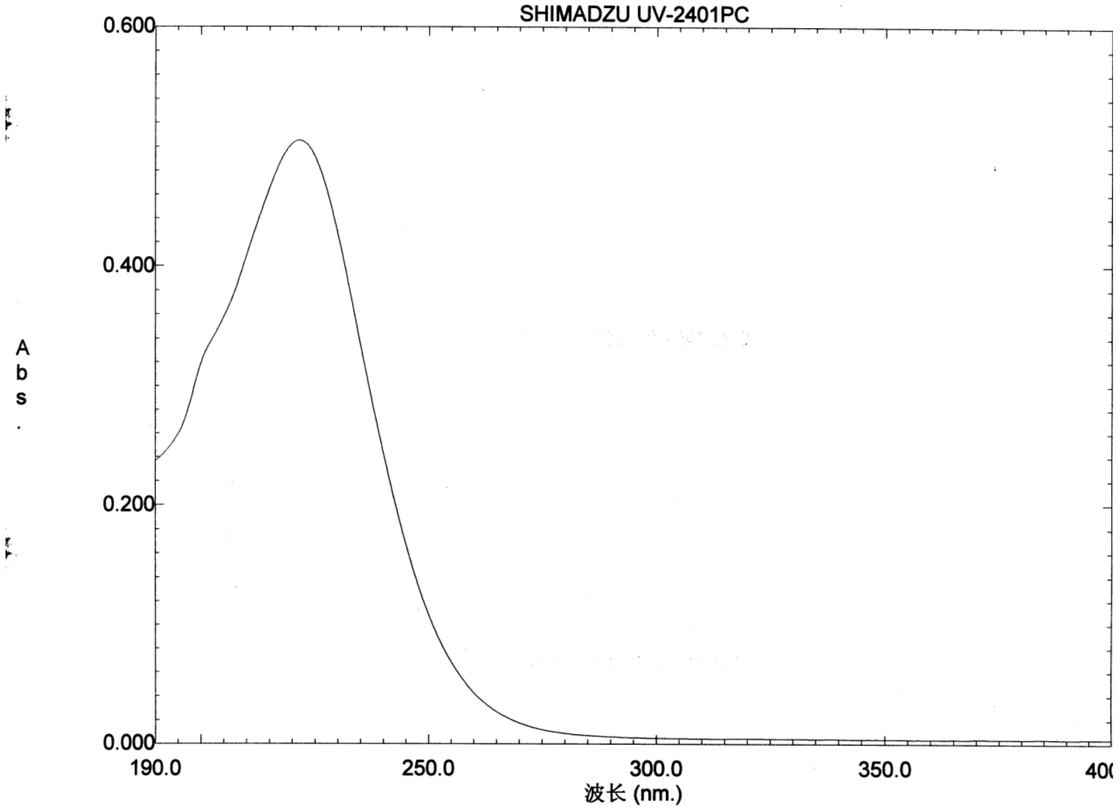

文件名: HXY51

HXY51 ———

样品浓度：0.0158毫克/毫升 → mol/L

溶剂：甲醇

创建于: 14:21 15-10-21

数据: 原始

测量模式: Abs.

扫描速度: 中速

狭缝: 5.0

采样间隔: 0.2

否.	波长 (nm.)	Abs.
1	221.60	0.5051

S10 UV spectrum of Bisebracteolasin A (1) in Methanol

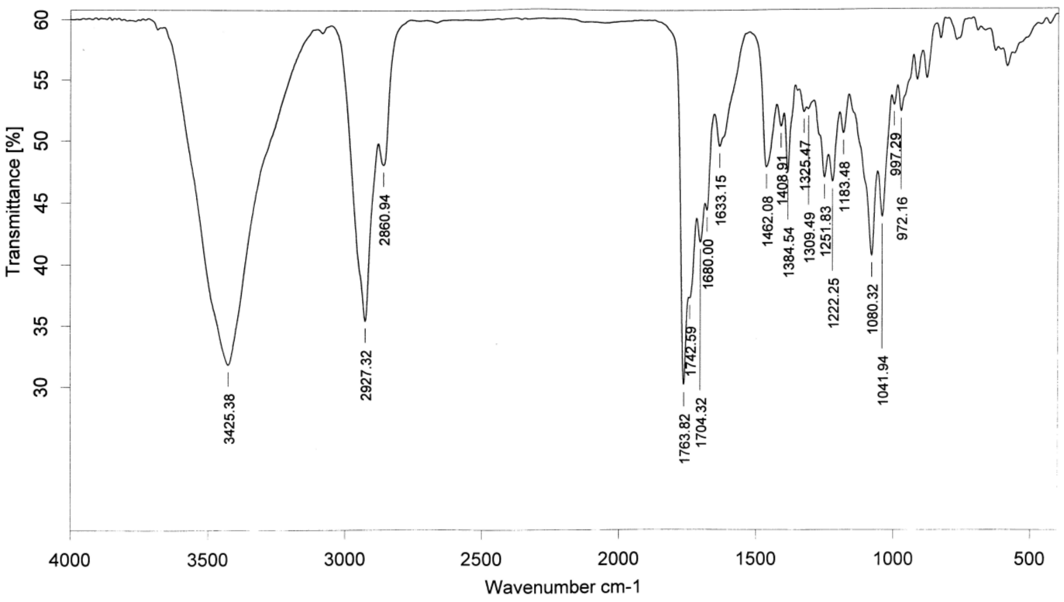

Sample : hyx51	Frequency Range : 399.246 - 3996.32		Measured on : 27/10/2015
Technique : KBr压片	Resolution : 4	Instrument : Tensor27	Sample Scans : 16
Customer : 151027IR1	Zerofilling : 2	Acquisition : Double Sided,For	

S11 IR spectrum of Bisebracteolasin A (1)

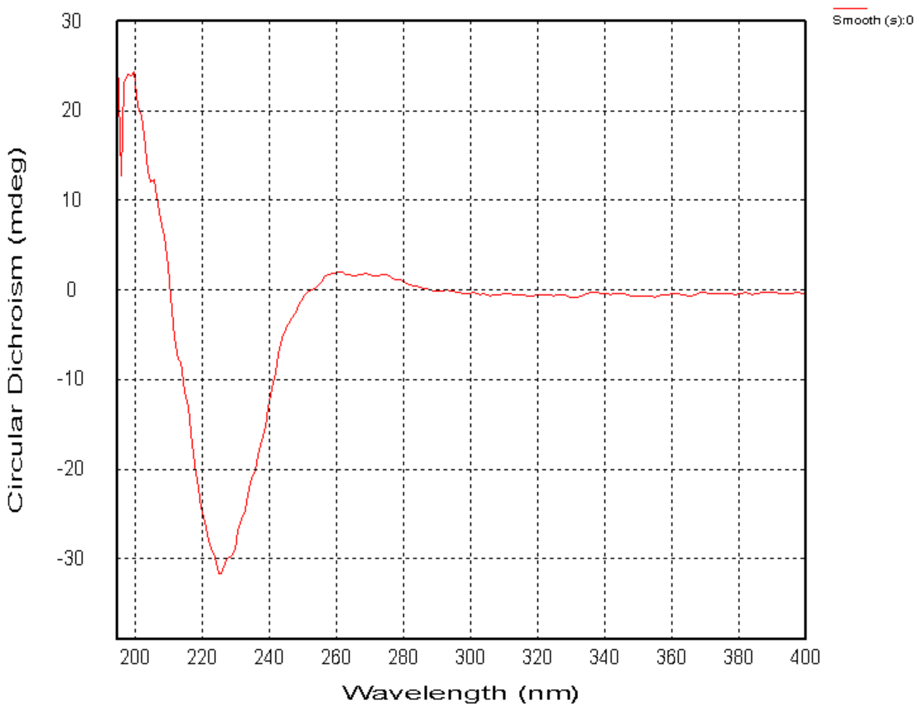

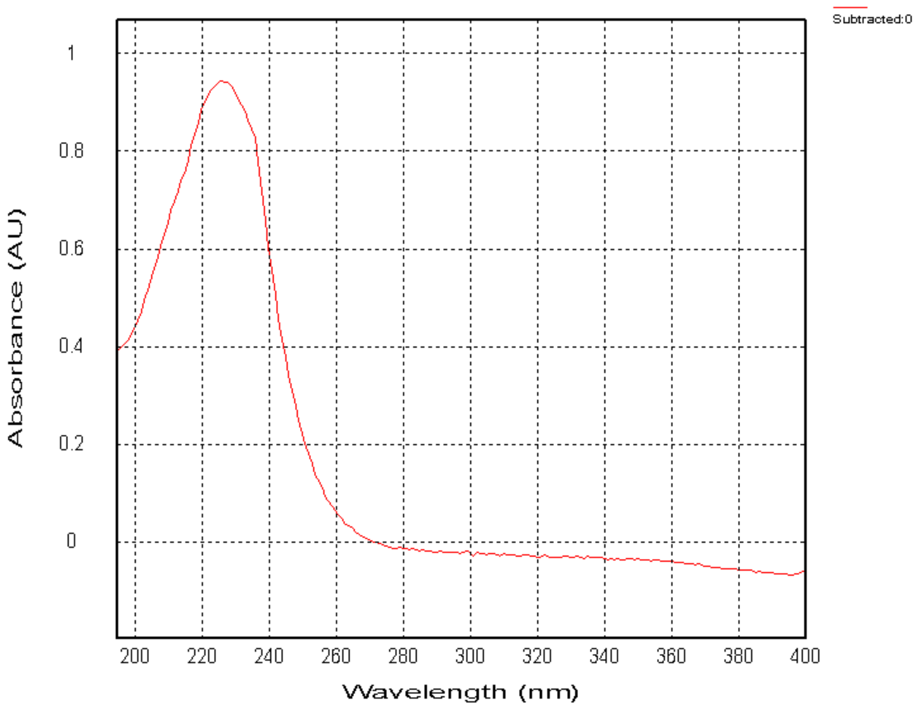

S12 CD spectrum of Bisebracteolasin A (1)

File: CD HYX51-1mm(195-400)17012310.dsx

ProBinaryX

Attributes :

- Time Stamp :Mon Jan 23 20:34:24 2017

- File ID : {52617AB3-225B-4077-9EBC-D136496DDA35}
- Is CFR Compliant : false
- Original unaltered data

Remarks:
  - HV (CDDC channel): 0 v
  - Time per point: 1 s
  - Description: Sample 1
  - Concentration: 0.4256 mg/mL MeOH
  - Pathlength: 1 mm

Settings:
  - Time-per-point: 1s (25us x 40000)
  - Wavelength: 195nm - 400nm
  - Step Size: 1nm
  - Bandwidth: 1nm

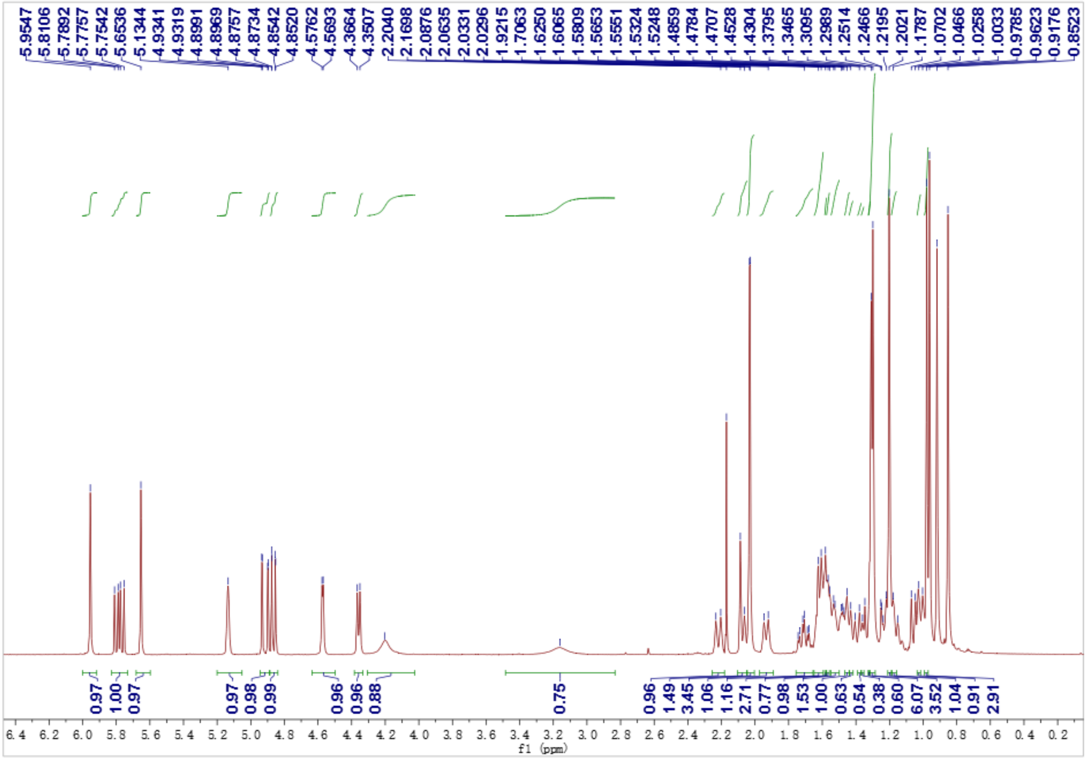

S13 ^{1}H NMR spectrum of Bisebracteolasin B (2) in $CDCl_3$-d_1

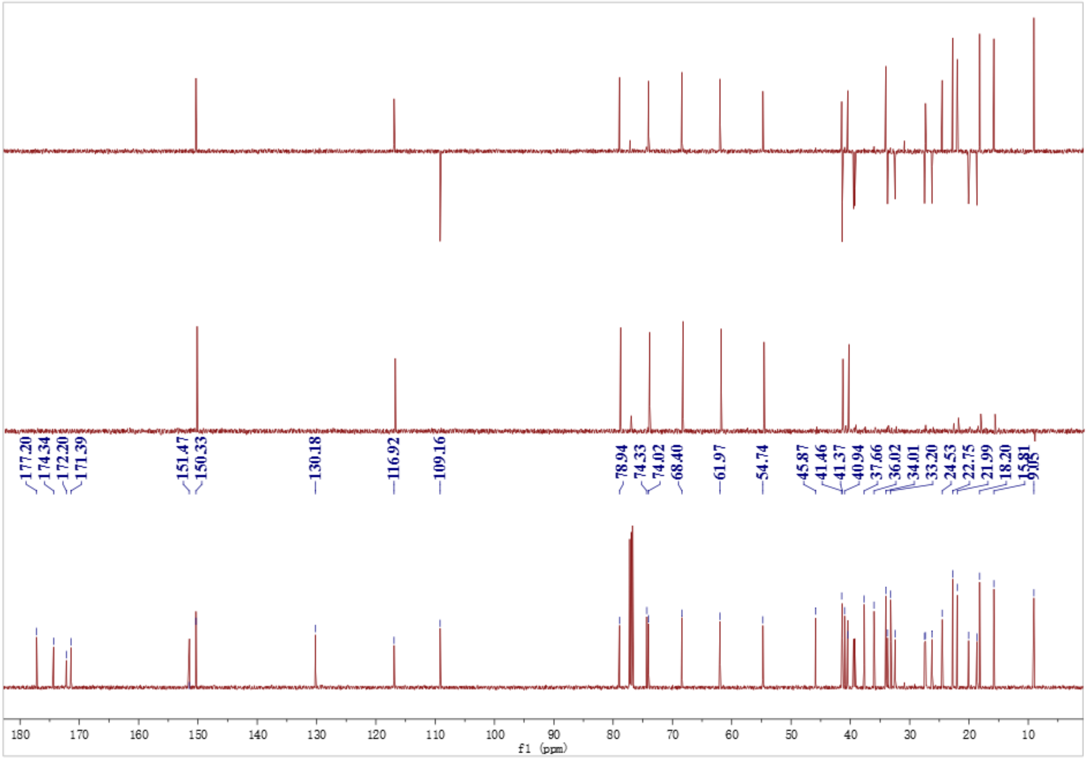

S14 ^{13}C NMR spectrum of Bisebracteolasin B (2) in $CDCl_3$-d_1

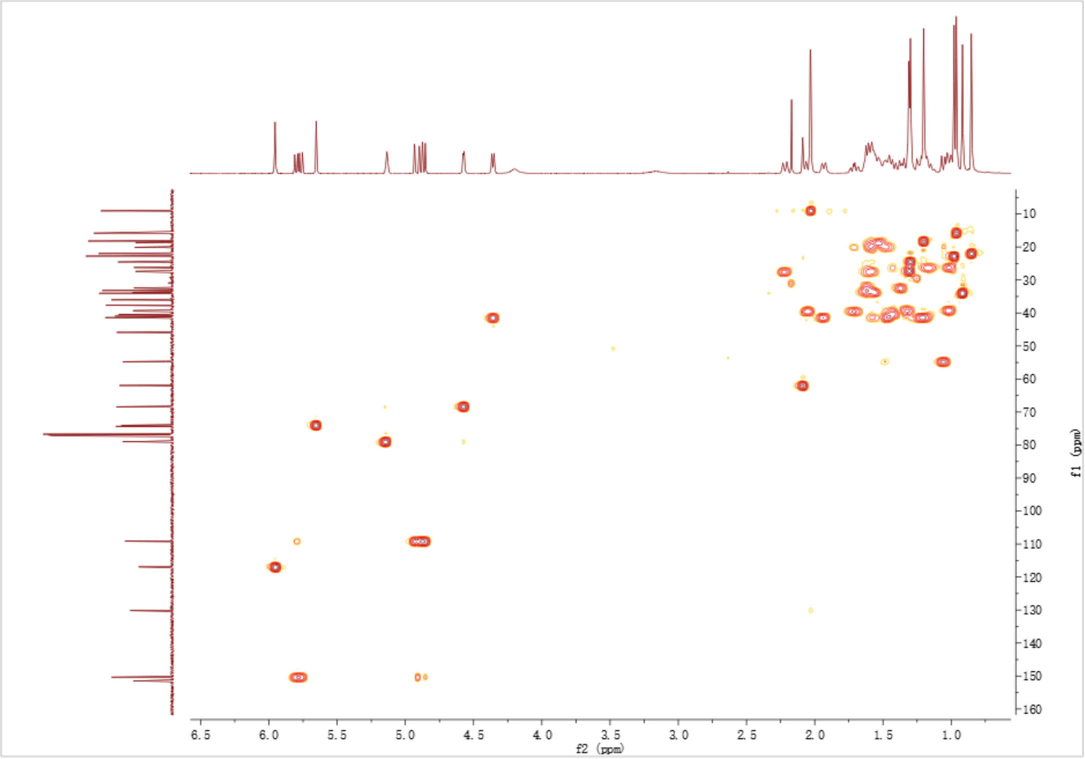

S15 HSQC spectrum of Bisebracteolasin B (2) in $CDCl_3$-d_1

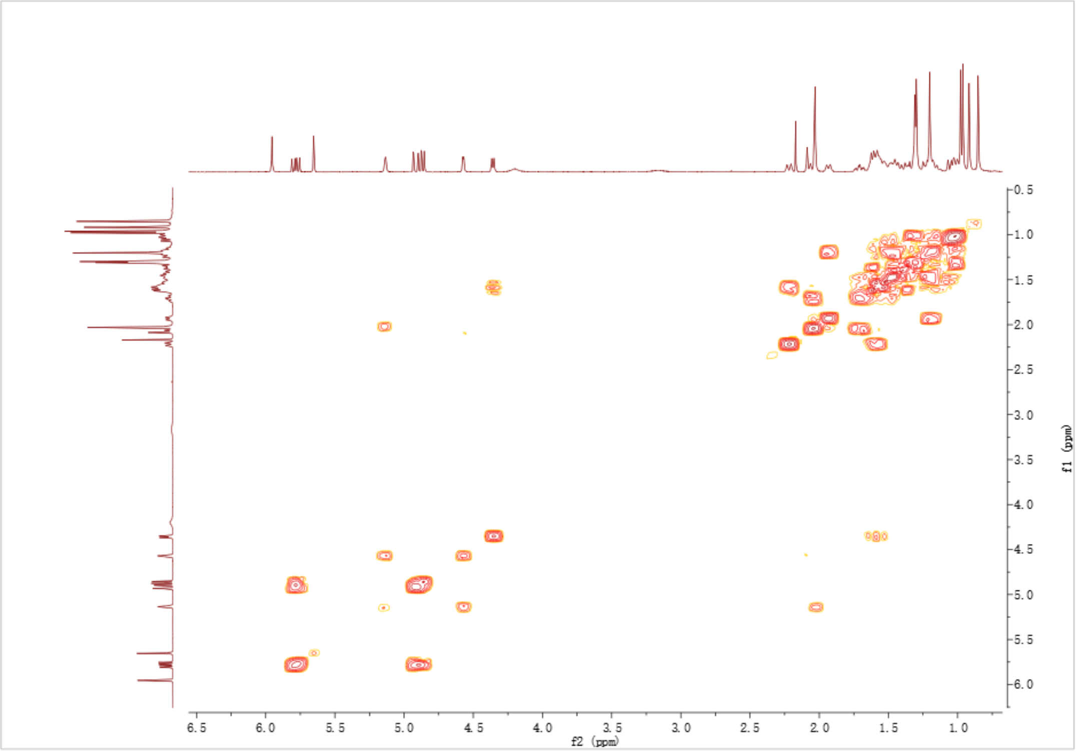

S16 COSY spectrum of Bisebracteolasin B (2) in $CDCl_3$-d_1

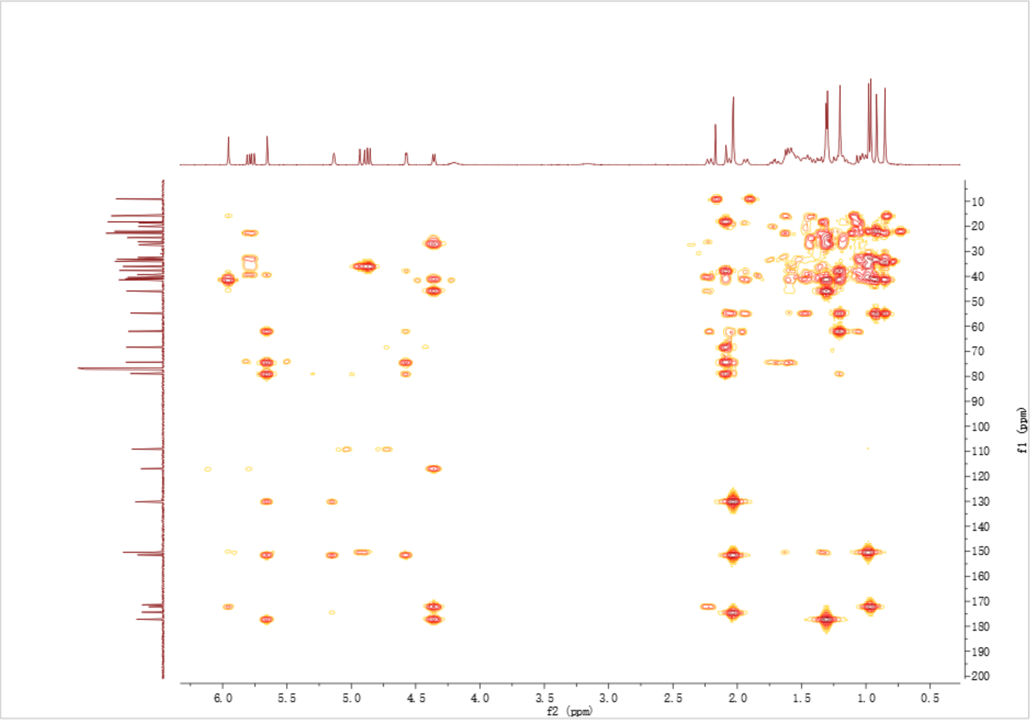

S17 HMBC spectrum of Bisebracteolasin B (2) in $CDCl_3$-*d*$_1$

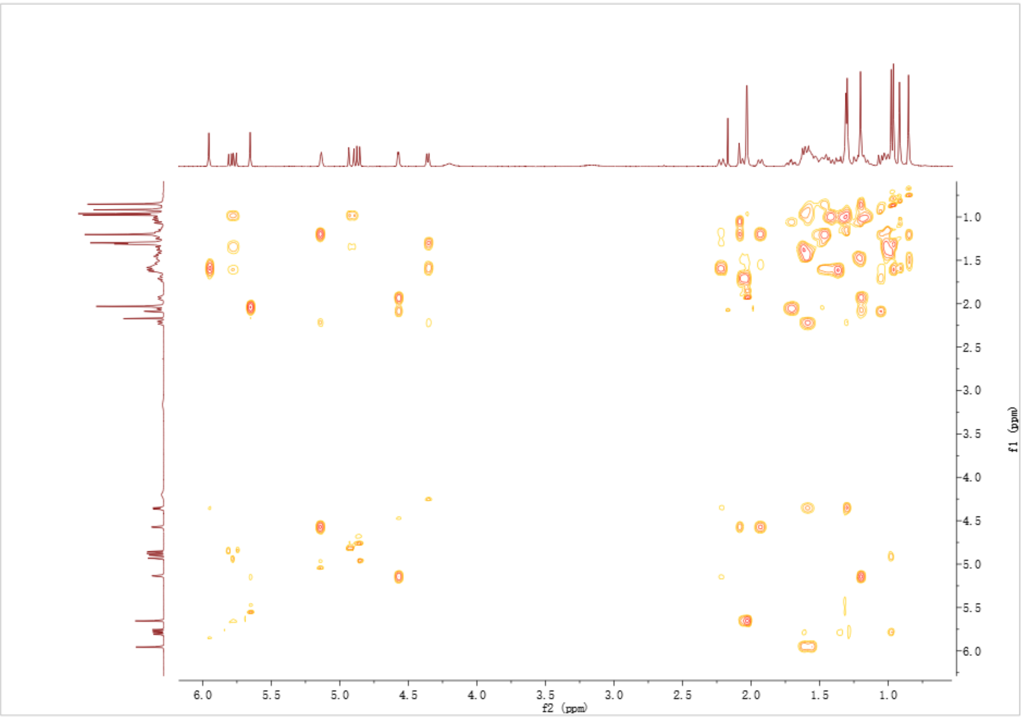

S18 ROESY spectrum of Bisebracteolasin B (2) in $CDCl_3$-*d*$_1$

Qualitative Analysis Report

**Data Filename**	hyx46.d	**Sample Name**	hyx46
Sample Type	Sample	**Position**	P1-B7
**Instrument Name**	Instrument 1	**User Name**	
**Acq Method**	SIBU.m	**Acquired Time**	10/14/2015 10:34:23 AM
**IRM Calibration Status**	Success	**DA Method**	ESI+.m
**Comment**			

Sample Group **Info.**

Acquisition SW 6200 series TOF/6500 series
Version Q-TOF B.05.01 (B5125.2)

User Spectra

Fragmentor Voltage	Collision Energy	Ionization Mode
135	0	ESI

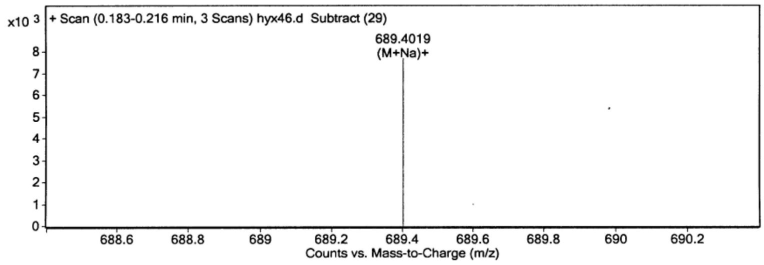

Peak List

m/z	z	Abund	Formula	Ion
274.2741	1	7858.6		
302.3063	1	2703.21		
318.3004	1	8765.95		
362.3265		2792.71		
684.4473	1	5172.6		
689.4019	1	7675.16	C40 H58 O8	(M+Na)+
690.4065	1	3165.63	C40 H58 O8	(M+Na)+
705.3745	1	3202.07		

Formula Calculator Element Limits

Element	Min	Max
C	3	60
H	0	120
O	0	30

Formula Calculator Results

Formula	CalculatedMass	CalculatedMz	Mz	Diff. (mDa)	Diff. (ppm)	DBE
C40 H58 O8	666.4132	689.4024	689.4019	0.1	0.2	12.0000

--- End Of Report ---

S19 HRESIMS spectrums of Bisebracteolasin B (2)

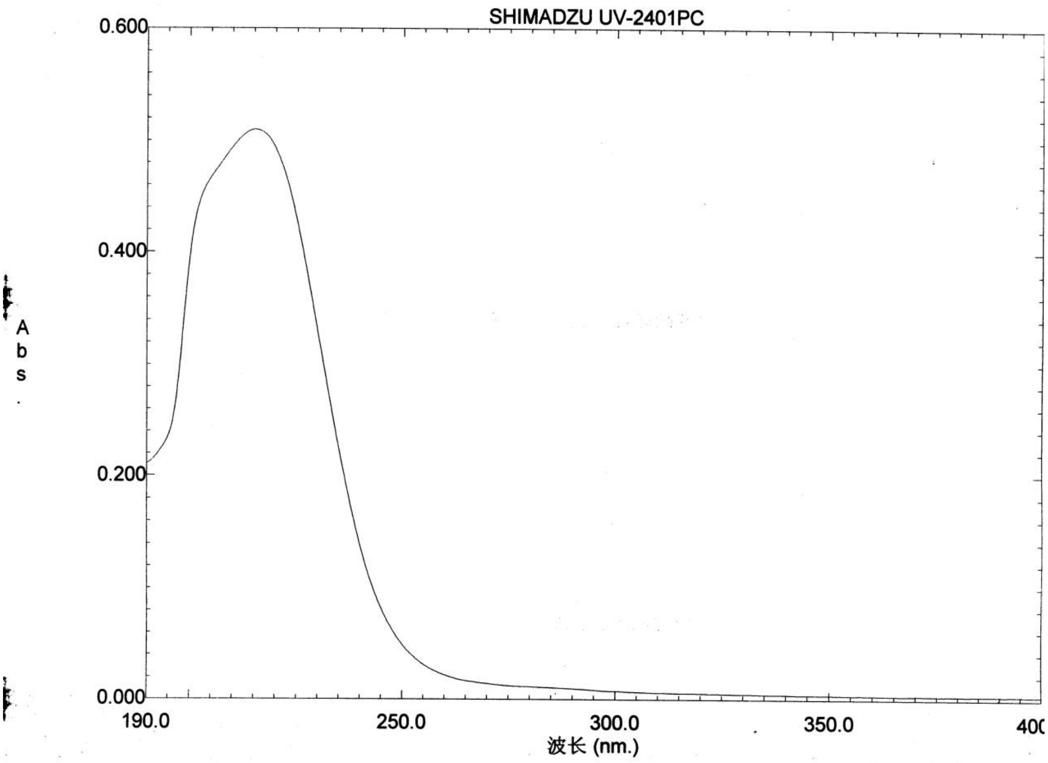

文件名:　HYX46

HYX46 ———

创建于:　14:07　15-10-21
数据:　原始

样品浓度：0.0156毫克/毫升
溶剂：甲醇

测量模式:　Abs.
扫描速度:　中速
狭缝:　5.0
采样间隔:　0.2

否.	波长 (nm.)	Abs.
1	215.40	0.5097

S20 UV spectrum of Bisebracteolasin B (2) in Methanol

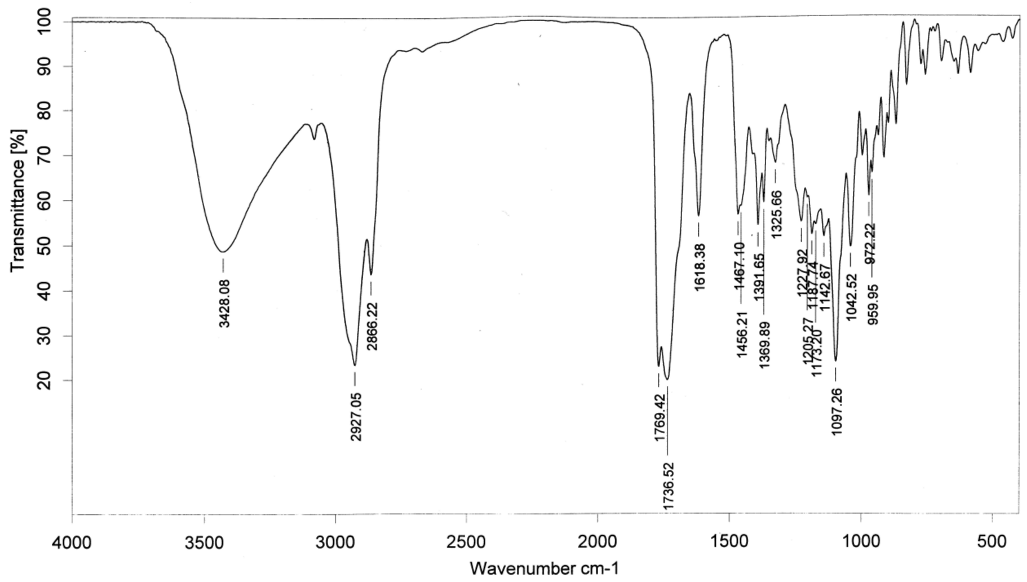

Sample : hyx46		Frequency Range : 399.246 - 3996.32	Measured on : 27/10/2015
Technique : KBr压片	Resolution : 4	Instrument : Tensor27	Sample Scans : 16
Customer : 151027IR0	Zerofilling : 2	Acquisition : Double Sided,For	

S21 IR spectrum of Bisebracteolasin B (2)

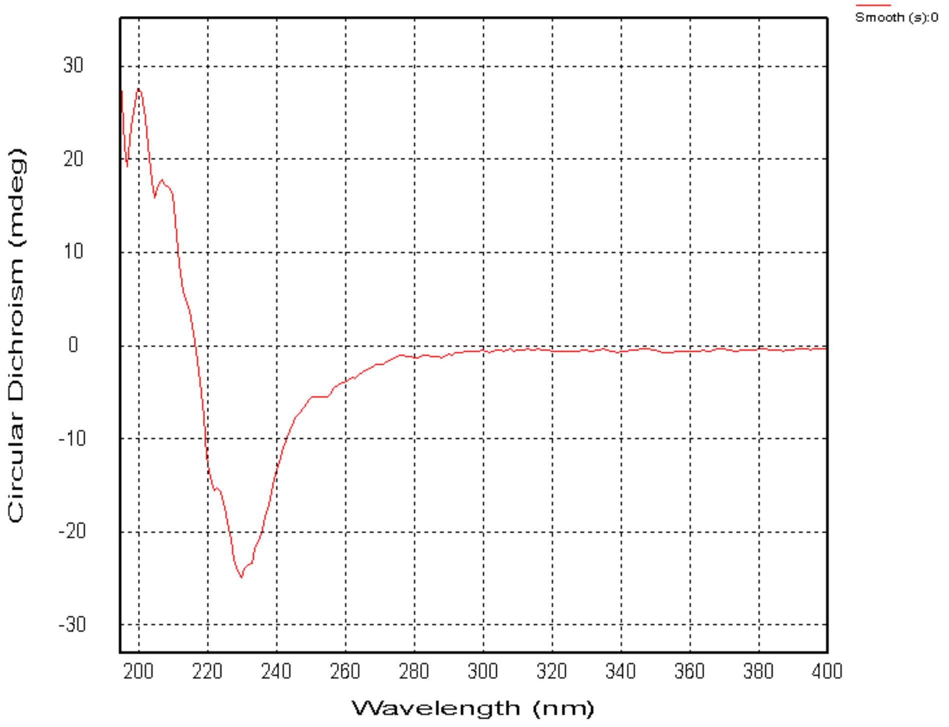

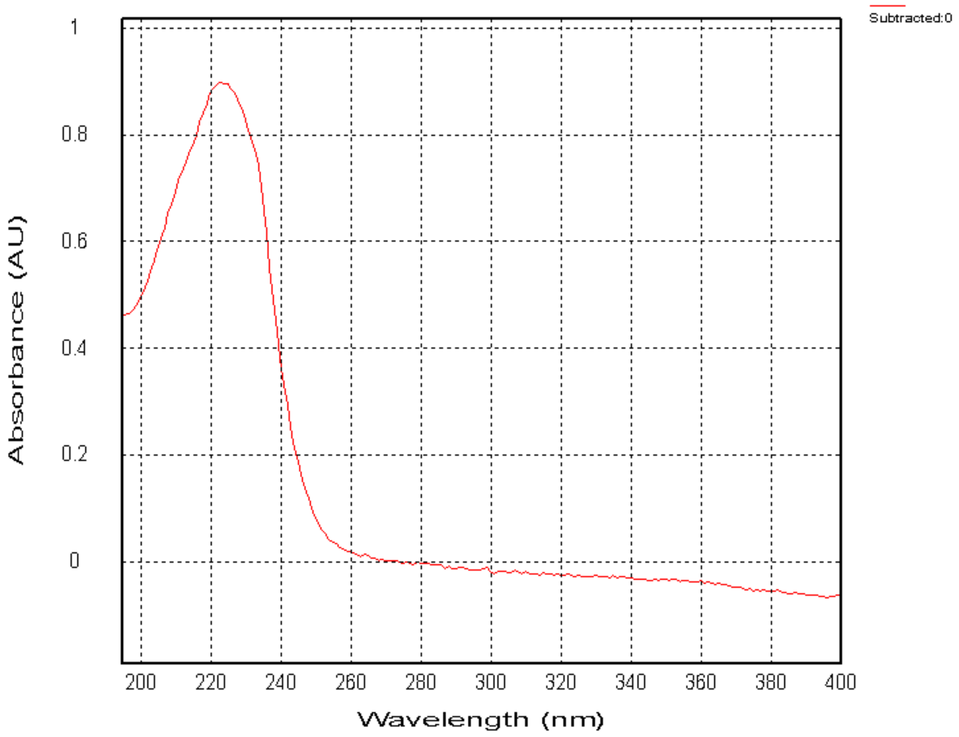

S22 CD spectrum of Bisebracteolasin B (2)

File: CD HYX46-1mm(195-400)17012309.dsx

ProBinaryX

Attributes :

- Time Stamp :Mon Jan 23 20:14:15 2017

- File ID : {ACBE4E12-8CEF-4e92-B362-9DEDC7794EF8}
- Is CFR Compliant : false
- Original unaltered data

Remarks:
  - HV (CDDC channel): 0 v
  - Time per point: 1 s
  - Description: Sample 1
  - Concentration: 0.2448mg/mL MeOH
  - Pathlength: 1 mm

Settings:
  - Time-per-point: 1s (25us x 40000)
  - Wavelength: 195nm - 400nm
  - Step Size: 1nm
  - Bandwidth: 1nm
